# An Attenuated CRISPR-Cas System in Enterococcus faecalis Permits DNA Acquisition

**DOI:** 10.1128/mBio.00414-18

**Published:** 2018-05-01

**Authors:** Karthik Hullahalli, Marinelle Rodrigues, Uyen Thy Nguyen, Kelli Palmer

**Affiliations:** a Department of Biological Sciences, The University of Texas at Dallas, Richardson, Texas, USA; Nanyang Technological University

**Keywords:** CRISPR-Cas, *Enterococcus faecalis*, genome editing, horizontal gene transfer

## Abstract

Antibiotic-resistant bacteria are critical public health concerns. Among the prime causative factors for the spread of antibiotic resistance is horizontal gene transfer (HGT). A useful model organism for investigating the relationship between HGT and antibiotic resistance is the opportunistic pathogen Enterococcus faecalis, since the species possesses highly conjugative plasmids that readily disseminate antibiotic resistance genes and virulence factors in nature. Unlike many commensal E. faecalis strains, the genomes of multidrug-resistant (MDR) E. faecalis clinical isolates are enriched for mobile genetic elements (MGEs) and lack clustered regularly interspaced short palindromic repeats (CRISPR) and CRISPR-associated protein (Cas) genome defense systems. CRISPR-Cas systems cleave foreign DNA in a programmable, sequence-specific manner and are disadvantageous for MGE-derived genome expansion. An unexplored facet of CRISPR biology in E. faecalis is that MGEs that are targeted by native CRISPR-Cas systems can be maintained transiently. Here, we investigate the basis for this “CRISPR tolerance.” We observe that E. faecalis can maintain self-targeting constructs that direct Cas9 to cleave the chromosome, but at a fitness cost. Interestingly, DNA repair genes were not upregulated during self-targeting, but integrated prophages were strongly induced. We determined that low *cas9* expression contributes to this transient nonlethality and used this knowledge to develop a robust CRISPR-assisted genome-editing scheme. Our results suggest that E. faecalis has maximized the potential for DNA acquisition by attenuating its CRISPR machinery, thereby facilitating the acquisition of potentially beneficial MGEs that may otherwise be restricted by genome defense.

## IMPORTANCE

CRISPR-Cas has provided a powerful toolkit to manipulate bacteria, resulting in improved genetic manipulations and novel antimicrobials. These powerful applications rely on the premise that CRISPR-Cas chromosome targeting, which leads to double-stranded DNA breaks, is lethal. In this study, we show that chromosomal CRISPR targeting in Enterococcus faecalis is transiently nonlethal. We uncover novel phenotypes associated with this “CRISPR tolerance” and, after determining its genetic basis, develop a genome-editing platform in E. faecalis with negligible off-target effects. Our findings reveal a novel strategy exploited by a bacterial pathogen to cope with CRISPR-induced conflicts to more readily accept DNA, and our robust CRISPR editing platform will help simplify genetic modifications in this organism.

## INTRODUCTION

Enterococcus faecalis is a Gram-positive opportunistic pathogen that is among the leading causes of hospital-acquired infections (1). E. faecalis is a natural colonizer of the human gastrointestinal tract, and frequent antibiotic usage promotes the proliferation of multidrug-resistant (MDR) strains. Intestinal overgrowth of MDR strains facilitates entry into the bloodstream, where complications like bacteremia and endocarditis can occur (2–4).

V583, the first reported vancomycin-resistant E. faecalis isolate in the United States, was isolated in 1987 from a bloodstream infection (5, 6). Further genomic characterization of V583 and other MDR strains led to the identification of several genetic characteristics that distinguished MDR isolates from commensal ones. Generally, MDR enterococci have larger genomes than commensal isolates, due to expanded collections of mobile genetic elements (MGEs). V583 possesses three plasmids (pTEF1 to -3), seven integrated prophages, and other MGEs (7, 8). MDR E. faecalis strains, including V583, also lack *cas* genes associated with clustered regularly interspaced short palindromic repeats (CRISPR) and CRISPR-associated protein (CRISPR-Cas) systems, which act as adaptive immune systems against bacteriophage and MGEs; genome defense is disadvantageous for horizontal acquisition of antibiotic resistance genes (9–11). However, commensal E. faecalis strains contain type II CRISPR-Cas systems, which have been extensively reviewed (12). Briefly, foreign DNA is first incorporated as a spacer in a repeat-spacer array (11, 13). The sequence in foreign DNA that is incorporated into the CRISPR array is known as the protospacer. The repeat-spacer array is transcribed into the pre-CRISPR RNA (pre-crRNA) and processed into short spacer-repeat fragments that form mature crRNAs (14, 15). A *trans*-encoded crRNA (tracrRNA) base pairs to the repeat region of the processed crRNA, and this dual-RNA complex associates with the Cas9 endonuclease (14, 16). The Cas9–dual-RNA complex surveys the genome for protospacer-adjacent motifs (PAMs) and, upon encountering a PAM that is immediately adjacent to the protospacer, cleaves the target DNA on both strands (17–22). Across the bacterial and archaeal domains, diverse CRISPR loci have been identified (reviewed in reference 12). Some CRISPR types possess alternative *cas* genes, cleave RNA targets, utilize different guides, and are otherwise mechanistically distinct from the type II CRISPR-Cas system we describe here (12).

MDR enterococci, which have arisen due to their propensity for acquiring antibiotic resistance genes, lack complete CRISPR systems (9). All E. faecalis strains, however, possess an orphan CRISPR locus, known as CRISPR2, that lacks *cas* genes (23). CRISPR1 and CRISPR3 are the functional CRISPR loci in E. faecalis, with a complete collection of type II *cas* genes upstream from the repeat-spacer array (24). Our previous work showed that integrating CRISPR1-*cas9* into V583, generating strain V649, restores the interference capability of CRISPR2 (25).

CRISPR-Cas has widely been used as a genome editing tool (26–30). CRISPR-assisted genome editing relies on the premise that targeting the chromosome, thereby inducing double-stranded DNA breaks (DSBs), is lethal and can select for outgrowth of low-frequency variants or rare recombinants (31). In our previous work, we described the perplexing ability for functional CRISPR-Cas and its targets to temporarily coexist in E. faecalis cells without compensatory mutations (25, 32). Rather than initially rejecting a CRISPR target, some E. faecalis cells transiently maintain it, but at a fitness cost. In the absence of selection, the CRISPR target is lost over time, while in the presence of selection, compensatory mutations (such as spacer loss or *cas9* inactivation) accumulate over time (25, 32). In this study, we generated a series of conjugative CRISPR-containing vectors that target the chromosome, and we show that E. faecalis can apparently survive simultaneous CRISPR-Cas9 targeting at multiple chromosomal locations. We show that chromosomal CRISPR targeting (also referred to as self-targeting) induces a transcriptional response distinct from the response to levofloxacin (LVX), a clinically relevant fluoroquinolone antibiotic. Robust induction of an apparent SOS response with LVX treatment and the concomitant lack of induction of these genes by CRISPR targeting led us to conclude that CRISPR self-targeting does not induce an SOS-like response in E. faecalis. However, CRISPR self-targeting induced the expression of all seven integrated prophages in V583. Finally, we demonstrate that increased expression of *cas9* leads to CRISPR lethality and contributes to bacteriophage resistance. We utilize this knowledge to develop a robust CRISPR-assisted genome-editing platform for E. faecalis. These findings, coupled with our previous results, reveal a mechanism used by a bacterial pathogen to overcome the limitations of possessing a genome defense system while preserving population-level protection against foreign DNA.

(This article was submitted to an online preprint archive [33].)

## RESULTS

### CRISPR self-targeting is not lethal in E. faecalis*.*

**FIG 1:**
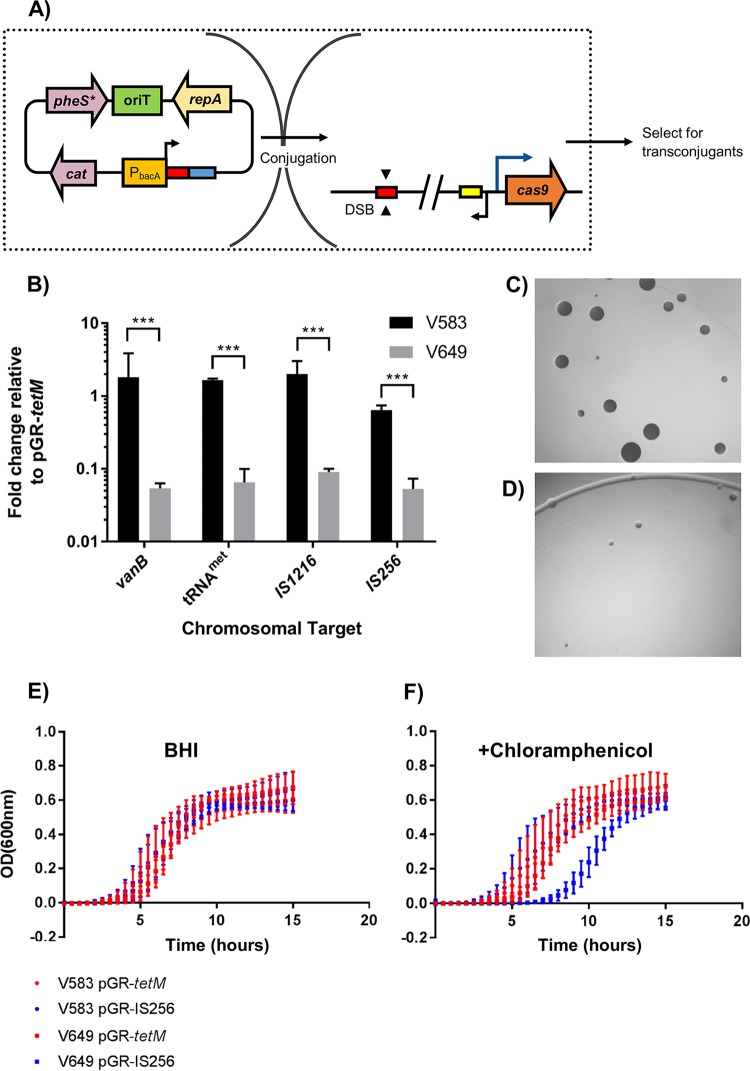
CRISPR tolerance protects against self-targeting. (A) Schematic diagram of conjugation experiments is shown. A donor strain carries a plasmid which encodes a CRISPR guide sequence (red rectangle), chloramphenicol resistance, and an origin of transfer. After conjugation, the crRNA guide associates with the Cas9 endonuclease (if present), which is chromosomally encoded in the recipient. The targeting complex then locates the protospacer (red rectangle; identical in sequence to spacer) on the recipient chromosome and cleaves the target sequence. The yellow rectangle represents the predicted tracrRNA, and the blue rectangle represents the repeat required for processing of the crRNA. Selection for transconjugants enumerates the number of recipient cells that accepted a self-targeting construct. (B) Conjugation frequencies expressed as transconjugants per donor relative to the frequency for pGR-*tetM* (control) are shown for plasmids that are predicted to generate 1 DSB (*vanB*), 5 DSBs (methionyl tRNA), 9 DSBs (IS*1216*), and 10 DSBs (IS*256*) (*n* = 3). Increasing the predicted number of DSBs does not further decrease conjugation frequency. (C) V649(pGR-*tetM*) (control) transconjugants on vancomycin and chloramphenicol selection media after 1 day of incubation are shown. (D) Same as described for panel C, but showing V649(pGR-IS*256*) (10 cuts) transconjugants. Chromosome targeting leads to an immediate growth defect on transconjugant selection medium. Pictures shown are at equal zoom. (E and F) OD_600_ values are shown for V583(pGR-*tetM*)/V649(pGR-*tetM*) (control) and V583(pGR-IS*256*)/V649(pGR-IS*256*) (10 predicted cuts) transconjugants grown in BHI (E) or BHI supplemented with chloramphenicol (F) (*n* = 2). V583 lacks *cas9*, while V649 possesses *cas9*. Chromosome targeting in V649 results in a growth defect in the presence of selection for the targeting plasmid. ***, *P* < 0.001.

We previously reported the ability of E. faecalis to transiently maintain CRISPR targets (25). It has also been postulated that CRISPR targets can be temporarily maintained through plasmid replication that proceeds faster than CRISPR targeting (34). To account for this possibility, the experiments in this study utilize vectors that direct Cas9 to target the chromosome; this ensures that CRISPR-Cas complexes would not need to compete with plasmid replication (Fig. 1A). To create a vector for facile generation of chromosome-targeting constructs, we modified a previously developed plasmid bearing a synthetic CRISPR that targeted *ermB* (25). We removed the first repeat upstream from the *ermB* spacer and introduced the promoter for pPD1 *bacA* (P_*bacA*_), which is strongly constitutive (35). Subsequently, we introduced pheS* to allow for counterselection on *para-*chloro-phenylalanine (*p*-Cl-Phe) (36). The resulting plasmid was designated pGR-*ermB* (GenBank accession number MF948287) and has advantages over its parent plasmid. In addition to counterselection, removal of the first repeat reduces the probability of spacer deletion while also allowing the spacer to be easily altered through PCR-directed mutagenesis (25). We subsequently modified the spacer to target different regions of the chromosome of E. faecalis V649 (V583 *cas9*) (25). We assumed that the number of instances where a protospacer target was present in the genome was proportional to the number of DSBs that would be caused via CRISPR self-targeting. We constructed four derivatives of pGR-*ermB* that were predicted to generate one DSB (targeting *vanB*, a gene for vancomycin resistance) or up to 10 DSBs (targeting the IS*256* transposase). A control predicted to generate no DSBs (pGR-*tetM*, targeting tetracycline resistance gene *tetM*, which is not present in V583) was also constructed. Consistent with our previous observations of CRISPR escape (25, 32), a large number of transconjugants arose despite chromosomal CRISPR targeting, and no change in conjugation frequencies was observed between pGR-*vanB* (1 DSB) and pGR-IS*256* (10 DSBs) (Fig. 1B). This suggested that total CRISPR lethality could not be achieved even with constructs that theoretically cleaved the genome in 10 distinct locations, in contrast to previous investigations of CRISPR self-targeting in other species (31, 37). This result was also observed in strain M236, an engineered derivative of Merz96 that encodes *cas9* (Fig. S1A), and strain OG1RF, which natively encodes the entire CRISPR1-Cas system (described later), demonstrating that this phenotype is not strain specific.

10.1128/mBio.00414-18.1 FIG S1  Chromosomal targeting in Merz96 and M236. Conjugation experiments identical to those described in the legend to Fig. 1 were performed. (A) Conjugation frequencies of pGR-*vanB* (1 predicted cut) relative to those of pGR-NPV1 (control) for Merz96 and M236 (Merz96 *cas9*) as recipients are shown as transconjugants per recipient (*n* = 5). (B and C) Merz96 or M236 (Merz96 *cas9*) transconjugants containing pGR-NPV1 or pGR-*vanB* were grown in BHI (B) or BHI supplemented with chloramphenicol (C), and OD_600_ was measured (*n* = 3). ***, *P* < 0.001. Download FIG S1, TIF file, 1 MB. Copyright © 2018 Hullahalli et al. 2018 Hullahalli et al. This content is distributed under the terms of the Creative Commons Attribution 4.0 International license.

Transconjugants of V649(pGR-IS*256*) were subsequently examined for phenotypic characteristics of this apparently nonlethal CRISPR self-targeting. Transconjugants that maintained CRISPR self-targeting constructs displayed slower colony growth than control constructs on medium with vancomycin (for selection of V649) or chloramphenicol (for selection of pGR-IS*256*) (Fig. 1C and D). Furthermore, V649(pGR-IS*256*) transconjugants possessed an extended lag phase in chloramphenicol broth relative to the lag phase of controls and were 2-fold more sensitive to LVX and ciprofloxacin (Fig. 1E and F; see Table S1 in the supplemental material). A growth defect was also observed in M236(pGR-*vanB*) transconjugants (Fig. S1B and C). These findings demonstrate that CRISPR self-targeting constructs confer deleterious but not lethal fitness effects on E. faecalis. We previously demonstrated that these phenotypes are associated with the transient maintenance of CRISPR conflicts without mutation of the CRISPR machinery in E. faecalis (25, 32).

10.1128/mBio.00414-18.7 TABLE S1  Fluoroquinolone MICs. Single transconjugant colonies were suspended in 5 ml BHI broth and used as inocula in broth microdilution antibiotic-susceptibility assays. Results are consistent across 3 replicates. Unit of concentration is µg/ml. Download TABLE S1, DOCX file, 0.01 MB. Copyright © 2018 Hullahalli et al. 2018 Hullahalli et al. This content is distributed under the terms of the Creative Commons Attribution 4.0 International license.

### Transcriptional responses to CRISPR- and fluoroquinolone-induced damage.

It is possible that CRISPR-Cas self-targeting in E. faecalis induces a robust SOS response as a consequence of DNA damage, which has been previously observed in Escherichia coli (38). To assess this hypothesis, we performed RNA sequencing to examine changes in gene expression due to CRISPR and LVX-induced damage. To assess CRISPR damage, strain V649(pGR-*tetM*) (control) and V649(pGR-IS*256*) (test) transconjugants from vancomycin/chloramphenicol selection were pooled and their RNA harvested. To assess LVX-induced damage, RNA was harvested from cultures prior to and 2 h after LVX administration at the MIC.

**FIG 2:**
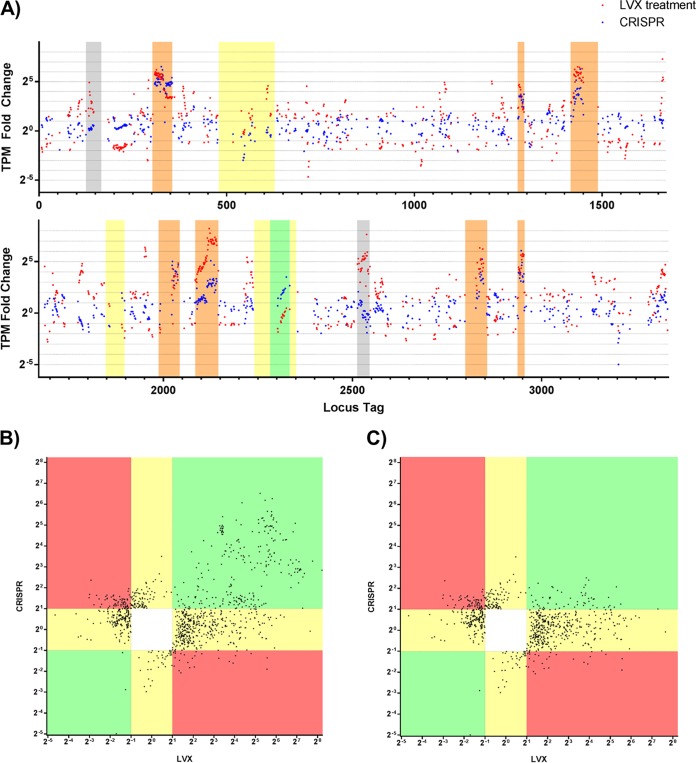
Transcriptomic responses to CRISPR-Cas9 self-targeting and LVX treatment. E. faecalis V649 was exposed to LVX or CRISPR self-targeting, and the corresponding changes in gene expression were measured via RNA sequencing. (A) Significant changes in gene expression across the V649 chromosome are plotted as the fold changes of transcripts per million (TPM) values for LVX (red dots) and CRISPR self-targeting (blue dots). Yellow, putative islands; grey, integrated plasmids; orange, prophages; green, vancomycin resistance transposon. See Data Set S1 in the supplemental material for full data set. (B) All genes (except those with fold changes of infinity) that were significantly (see Materials and Methods) differentially regulated by either LVX or CRISPR treatment were plotted, irrespective of individual *P* value. The horizontal axis represents the fold change of gene expression caused by LVX, and the vertical axis represents the corresponding fold change of gene expression caused by CRISPR self-targeting. Green regions indicate genes that were similarly differentially regulated by CRISPR and LVX. Red regions indicate genes that were oppositely differentially regulated by CRISPR and LVX. Yellow regions indicate genes that were differentially regulated by either CRISPR or LVX but not both. The white region lacks data points since these would correspond to genes that were not differentially regulated by either CRISPR or LVX, and these genes were statistically filtered out. (C) Same as described for panel B except lacking genes located on prophage elements.

**FIG 3:**
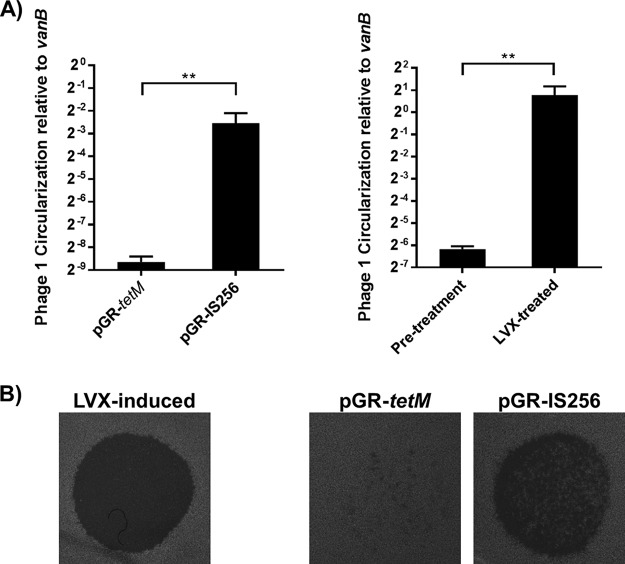
CRISPR self-targeting induces prophages. (A) Results of quantitative PCR (qPCR) on genomic DNA harvested from cultures treated by CRISPR or LVX are shown (*n* = 3). Phage01 circularization, indicating excision from the chromosome, was normalized to abundance of *vanB*. Both CRISPR targeting and levofloxacin induced phage circularization. (B) Undiluted filtrates of supernatants from E. faecalis cultures were spotted on lawns of E. faecalis ATCC 29212. Infectious phage particles were detected in cultures with CRISPR self-targeting. For these experiments, cultures were treated identically to those prepared for transcriptomics analysis as described in Materials and Methods. **, *P* < 0.01.

After statistical filtering, 999 genes in V649 were significantly differentially expressed in response to either LVX treatment or CRISPR self-targeting (Data Set S1). Two hundred twenty-seven genes were significantly upregulated during CRISPR self-targeting, and 626 were significantly upregulated by LVX, with 162 genes upregulated under both conditions. Therefore, 71.4% of genes upregulated during CRISPR self-targeting were also upregulated by LVX, but only 25.9% of genes upregulated by LVX were also upregulated by CRISPR (Fig. 2). Prophage genes were upregulated by both CRISPR and LVX. Seventy percent of the genes that were significantly upregulated by CRISPR self-targeting alone were located in prophage elements. Increases in circular Phage01 DNA and infectious phage particles were detected in LVX and CRISPR treatments (Fig. 3). This correlates well with observations of prophage induction upon ciprofloxacin exposure (39). Importantly, induction of canonical features of the SOS DNA damage response, including *recA*, *dinP*, and EF1080 (predicted *umuC*), was observed with LVX treatment but not with CRISPR self-targeting (Data Set S1) (40). Furthermore, various regions of the genome were regulated discordantly between our two experimental conditions. LVX treatment upregulated genes on two integrated plasmids, but CRISPR did not. Interestingly, a cluster of genes in the vancomycin resistance transposon were upregulated by CRISPR but not differentially regulated by LVX (Fig. 2). Collectively, these data demonstrate that E. faecalis responds to CRISPR self-targeting in a manner distinct from a fluoroquinolone-induced stress response. Taken together with our previous findings, we directly demonstrate a unique phenotype associated with CRISPR targeting in E. faecalis, characterized by prophage induction but no canonical DNA damage response. We hereinafter refer to this transient maintenance of CRISPR targets and the corresponding phenotypes as “CRISPR tolerance.”

10.1128/mBio.00414-18.10 DATA SET S1  Changes in gene expression resulting from CRISPR self-targeting and LVX treatment. The fold changes of gene expression in response to LVX (FC-LVX) and CRISPR (FC-CRISPR) treatment are indicated for all genes that were differentially regulated as described in “Transcriptomics analysis” in the text. Also included are sheets which categorize genes up- and downregulated by CRISPR or LVX treatment. For these sheets, if the fold change of gene expression was >2 but the *P* value was >0.05, a fold change of 1 was manually entered; the true fold change value can be found in the master sheet. Download DATA SET S1, XLSX file, 0.2 MB. Copyright © 2018 Hullahalli et al. 2018 Hullahalli et al. This content is distributed under the terms of the Creative Commons Attribution 4.0 International license.

### Genetic basis for CRISPR tolerance.

**FIG 4:**
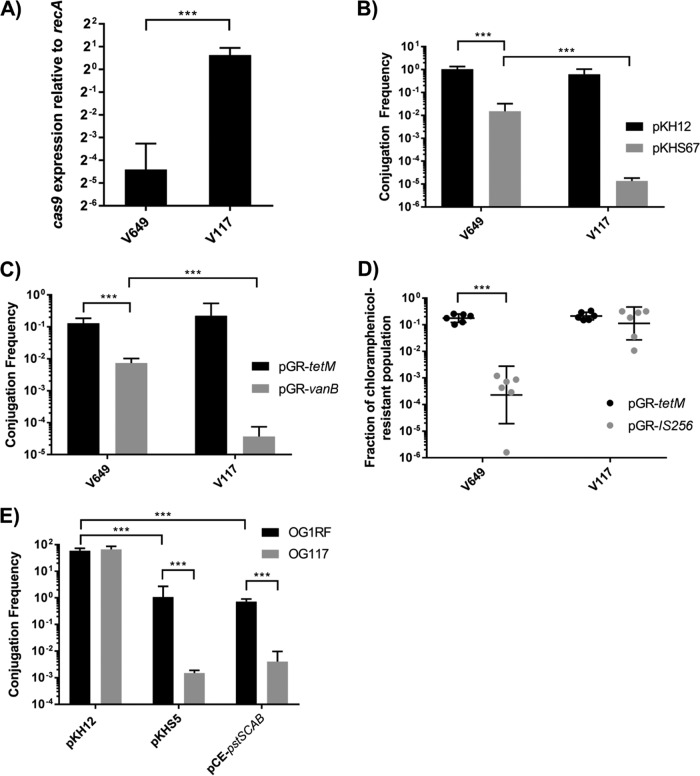
Low *cas9* expression is the genetic basis for CRISPR tolerance. (A) *cas9* expression was measured by RT-qPCR for V649 (V583 *cas9*) and V117 (V583 P_*bacA*_-*cas9*), thereby verifying that P_*bacA*_ increases the expression of *cas9* (*n* = 3). Expression was normalized to that of *recA*. (B) Conjugation frequencies of pKH12 (control) and pKHS67 (protospacer target for S67 on the V583 chromosome) into V649 (V583 *cas9*) and V117 (V583 P_*bacA*_-*cas9*) are shown as transconjugants per donor (*n* = 3). (C) Conjugation frequency of a control (pGR-*tetM*) or a chromosomal CRISPR targeting plasmid (1 cut, pGR-*vanB*) is shown as transconjugants per donor (*n* = 3). (D) Plasmid retention, as fraction of chloramphenicol-resistant population, is shown for pGR-*tetM* and pGR-IS*256* in V649 (V583 *cas9*) and V117 (V583 P_*bacA*_-*cas9*) populations passaged for 2 days in the absence of chloramphenicol selection. CRISPR-specific plasmid loss is a hallmark of CRISPR tolerance, and transconjugants possessing increased *cas9* expression do not display this phenotype. (E) Conjugation frequencies of pKH12 (control), pKHS5 (targeted by CRISPR2 of OG1RF), and pCE-*pstSCAB* (targets chromosome for CRISPR editing) for OG1RF and OG117 (OG1RF P_*bacA*_-*cas9*) recipients are shown as transconjugants per donor (*n* = 3). This confirms that increasing *cas9* expression overcomes CRISPR tolerance in E. faecalis strains other than V583. The limit of detection was 1,000 CFU/ml for all panels. *, *P* < 0.05; ***, *P* < 0.001.

We hypothesized that increasing the abundance of certain components of the CRISPR machinery would potentiate CRISPR chromosome targeting and lead to lethality. We introduced P_*bacA*_ upstream from *cas9* and examined the conjugation frequencies of CRISPR-targeted plasmids. Twenty-seven-fold upregulation of *cas9* was verified with reverse transcription quantitative PCR (RT-qPCR) (Fig. 4A). We previously showed that pKHS67, targeted by spacer 67 on the V649 CRISPR2 locus, possesses markedly reduced conjugation frequencies relative to those of pKH12, which lacks a protospacer target (25). When *cas9* expression is increased (strain V117 [V583 P_*bacA*_-*cas9*]), a significantly greater reduction in conjugation frequency is observed, and pKHS67 transconjugants fall to near or below the levels of detection (Fig. 4B). Similarly, we observe very few V117 transconjugants that arise from chromosomal targeting with pGR-*vanB* (Fig. 4C). We then hypothesized that the few V117 transconjugants that accepted CRISPR targets were mutants with inactivated CRISPR-Cas. To investigate this, we assessed plasmid maintenance in the absence of selection. Our previous data showed that CRISPR-dependent plasmid loss in the absence of selection is one of the phenotypes of CRISPR tolerance (25, 32). Expectedly, V649(pGR-IS*256*) transconjugants demonstrate marked plasmid loss after 2 days of passaging without selection, characteristic of the CRISPR tolerance phenotype and consistent with pGR-IS*256* conferring a fitness defect to host cells (Fig. 4D). However, V117(pGR-IS*256*) transconjugants on average show no significant plasmid loss, indicating that these are true CRISPR mutants (Fig. 4D). We verified that these observations extend to E. faecalis strains natively bearing *cas9* by investigating OG1RF, which natively possesses the functionally linked CRISPR1-Cas and CRISPR2 loci. Consistent with results obtained in V649 and M236, we observed a 2-log reduction in conjugation frequency with pKHS5, which is targeted by S5 on the OG1RF CRISPR2 locus, relative to the conjugation frequency in the control. We then inserted P_*bacA*_-*cas9* into OG1RF, creating strain OG117. We observed a significant, 5 log reduction in conjugation frequency for pKHS5 relative to the conjugation frequency of pKH12 in OG117. The conjugation frequency of a chromosome-targeting construct, pCE-*pstSCAB* (described below), was similarly reduced in OG117 (Fig. 4E). These results collectively demonstrate that increased *cas9* expression overcomes CRISPR tolerance and results in CRISPR lethality and implicate low *cas9* expression as the genetic basis for CRISPR tolerance.

**FIG 5:**
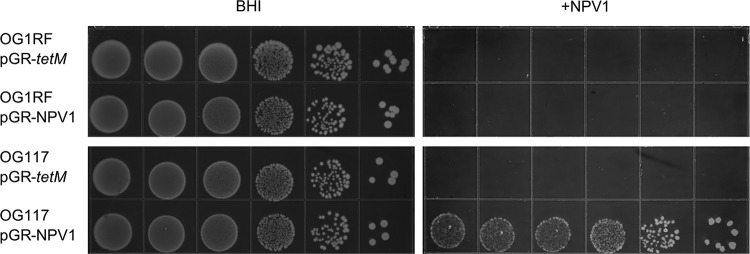
Native *cas9* expression does not protect against bacteriophage. OG1RF and OG117 (OG1RF P_*bacA*_-*cas9*) containing either pGR-*tetM* (control) or pGR-NPV1 (targets ΦNPV1) were spotted on BHI or BHI with ΦNPV1 in a soft agar overlay. The appearance of colonies on plates possessing ΦNPV1 indicates phage resistance, which is achieved only when *cas9* expression is increased and the correct guide sequence is present. Chloramphenicol was included to promote plasmid maintenance. Results for 10-fold dilutions are shown. Results were consistent across 3 biological replicates.

We also investigated whether *cas9* expression contributed to phage resistance, since one of the most well-characterized functions of CRISPR-Cas is antiphage defense (10). We designed pGR-NPV1, which targets ΦNPV-1, a phage that infects OG1RF (41). We exposed cultures of OG1RF and OG117 containing either pGR-*tetM* (control) or pGR-NPV1 to ΦNPV1. OG1RF was sensitive to ΦNPV-1 even when possessing pGR-NPV1. However, OG117 (OG1RF P_*bacA*_-*cas9*) was resistant to ΦNPV-1 when it possessed pGR-NPV1 but not pGR-*tetM* (Fig. 5). These results demonstrate that native *cas9* expression under routine laboratory conditions is not sufficient to confer defense against phage in E. faecalis.

### CRISPR-assisted genome editing in E. faecalis*.*

Knowing that *cas9* overexpression leads to lethality of CRISPR self-targeting, we sought to develop an efficient CRISPR-mediated editing scheme for E. faecalis, since none had been reported. We modified pGR-*vanB* to encode a homologous recombination template which conferred a 100-bp deletion of *vanB* (Fig. S2A). Successful edits would abolish vancomycin resistance and therefore allow us to utilize a rapid screen. The new plasmid, designated pCE-*vanB*, was conjugated into strains V649 (V583 *cas9*) and V117 (V583 P_*bacA*_-*cas9*), and transconjugants were selected on erythromycin (for V649 or V117 selection) and chloramphenicol (for pCE-*vanB* selection). After 2 days, V117 transconjugant colonies appeared at low frequencies. Interestingly, two colony morphologies were observed for V649 transconjugants; some were large and appeared after 2 days, but most were slower growing and apparent after 3 days. We distinguished these phenotypes as “early” (the larger colonies) and “late” (the smaller colonies). Transconjugants from all three groups (V117, V649 early, and V649 late) were restruck on chloramphenicol agar and then screened for vancomycin sensitivity. Remarkably, 83% of V117 transconjugants were vancomycin sensitive. Fifty percent of the early V649 transconjugants and 22% of the V649 late transconjugants were vancomycin sensitive (Table 1). The restreak on chloramphenicol was essential for CRISPR-assisted editing of *vanB*, as V117(pCE-*vanB*) transconjugant colonies on the initial erythromycin/chloramphenicol selection still possessed some cells that were vancomycin resistant (Fig. S2B). Vancomycin-sensitive clones were passaged and plated on counterselective medium to identify clones that lost pCE-*vanB*, and these were screened for the desired edit by PCR (Fig. S2C). All vancomycin-sensitive clones that were PCR screened contained a 100-bp deletion of *vanB*. Editing in V649 reveals that homologous recombination can rescue these cells from the effects of CRISPR tolerance, albeit at markedly lower efficiencies than when *cas9* is overexpressed (Table 1).

10.1128/mBio.00414-18.2 FIG S2  CRISPR editing of *vanB*. (A) Plasmid schematic and PCR screening primers are shown. (B) Twelve initial V117(pCE-*vanB*) transconjugant colonies were resuspended in PBS and plated on selective and nonselective agar to quantify vancomycin-resistant and total colonies as measured in CFU. The presence of a vancomycin-resistant subpopulation indicates the heterogeneous nature of the initial transconjugant colony, where a fraction of cells still remain unedited. (C) Representative CRISPR editing in vancomycin-sensitive clones obtained after passaging (to resolve heterogeneity from the experiment whose results are shown in panel B) and counterselection. Edited products are 100 bp smaller than unedited products. CRISPR2 was amplified as a control to verify that edited clones are not donor strains, which possess a longer CRISPR2 array than V117 and V649 (23). Download FIG S2, TIF file, 1 MB. Copyright © 2018 Hullahalli et al. 2018 Hullahalli et al. This content is distributed under the terms of the Creative Commons Attribution 4.0 International license.

**TABLE 1:** CRISPR editing experiments performed in this study^a^

Strain	Edit	Mean editing efficiency ± SD (%)^b^	Type of edit	Size of edit (kb)
V649 early	*vanB*	50 ± 34.8	Deletion	0.1
V649 late	*vanB*	21.8 ± 18.3	Deletion	0.1
V117	*vanB*	83.3 ± 13.6	Deletion	0.1
	*pstB*	66.7 ± 16.7	Deletion	0.8
	*pstSCAB*	55.6 ± 9.6	Deletion	4.3
	EF3217		Deletion	2.9
V200	*pstSCAB*	77.8 ± 9.6	Deletion	4.3
	*tetM*	38.8 ± 9.6	Insertion	2.5
OG117	*pstSCAB*	94.4 ± 9.6	Deletion	4.3

a CRISPR editing of *vanB* was screened phenotypically, while the products of all other experiments were screened by PCR. *tetM* was inserted between EF1866 and EF1867. For editing of *vanB*, two colony morphologies were observed on the initial transconjugant selection (vancomycin and chloramphenicol); “early” colonies arose after 2 days, while “late” colonies arose after 3 days.

b Editing efficiency was calculated as the number of successful edits as a percentage of the total number of clones screened. Each experiment was performed in at least biological triplicate. Six clones were screened in each replicate. The exception was the deletion of EF3217, which was performed solely to generate the mutant.

**FIG 6:**
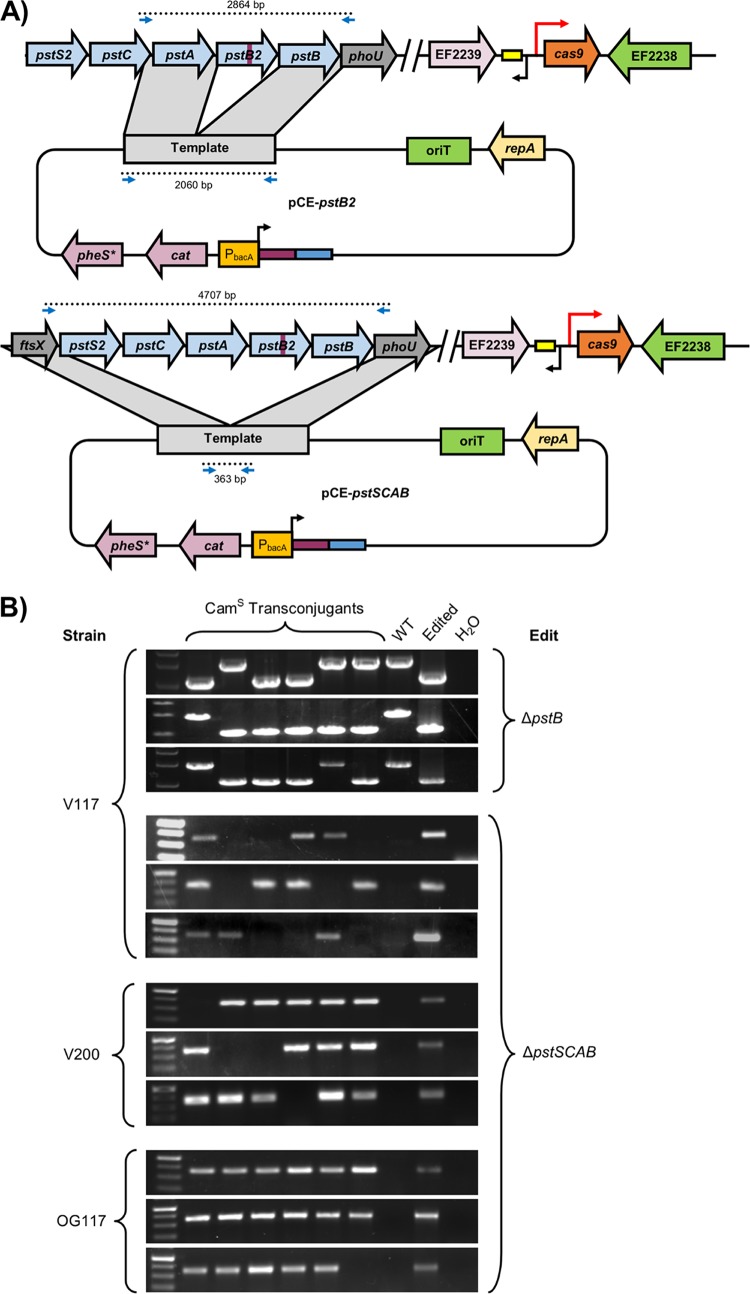
CRISPR editing in E. faecalis. (A) Plasmids, editing schematic, and screening primers are shown for deletions of *pstB* and *pstSCAB*. The purple rectangle represents the spacer, and the blue rectangle represents the repeat. (B) Editing experiments are shown for individual experiments (three per group) in V117 (V583 P_*bacA*_-*cas9*), V200 (V583 P*_bacA_-cas9* ΔEF3217), and OG117 (OG1RF P_*bacA*_-*cas9*) with indicated edits. PCR was performed to examine the edited locus for the desired modification. Frequencies are shown in Table 1. Successful edits and appropriate negative controls are shown as indicated. All clones were verified to be chloramphenicol sensitive, indicative of plasmid loss.

To further evaluate the efficiency of CRISPR-assisted editing, we designed a construct to delete genes encoding the putative phosphate transporter *pstB2* or the entire operon consisting of *pstS2*, *pstC*, *pstA*, *pstB2*, and *pstB* (here referred to as *pstSCAB*) (Fig. 6A). Sixty-seven percent and 56 percent of V117 clones screened by PCR had deletions in *pstB* and *pstSCAB*, respectively. Furthermore, *pstSCAB* deletion by CRISPR editing was highly efficient in strain OG117 (strain OG1RF P_*bacA*_-*cas9*) (95% editing success), demonstrating that CRISPR-assisted editing can be achieved in different E. faecalis strains (Fig. 6B).

During these experiments, the conjugation frequency of chromosomal CRISPR targeting constructs into V117 (V583 P_*bacA*_-*cas9*) was low; only ~100 CFU/ml transconjugants were obtained in some experiments. We sought a method to increase conjugation frequency and avoid plating extremely high cell densities to detect modified clones. The New England Biolabs REBASE (42) predicted a type IV restriction endonuclease in V583 (EF3217), for which a homologue was biochemically assessed in Staphylococcus aureus (43). The predicted recognition site (SCNGS) from S. aureus corresponded to known 5-methylcytosine methylation sites in the E. faecalis OG1 derivatives OG1RF and OG1SSp (G^m5^CWGC) (44). Since the donor used for conjugation in our experiments is also derived from OG1, we hypothesized that deletion of EF3217 in the recipient would increase the conjugation frequencies of CRISPR editing constructs. We therefore generated strain V200, a V117 derivative which lacks EF3217, using CRISPR-assisted editing. The conjugation frequencies of all plasmids, even those targeting the chromosome, were significantly greater for V200 recipients than for V117 recipients (Fig. S3). We also successfully performed CRISPR-assisted editing in V200 (V583 P*_bacA_-cas9* ΔEF3217), demonstrating that successive CRISPR edits are possible in our system (Fig. 6B and Table 1).

10.1128/mBio.00414-18.3 FIG S3  Deletion of EF3217 increases conjugation frequency. pGR-*tetM* (control), pGR-*vanB* (targets chromosome), and pCE-*pstSCAB* (used for CRISPR editing) were conjugated into V117 (V583 P_*bacA*_-*cas9*) or V200 (V583 P_*bacA*_-*cas9* ΔEF3217), and conjugation frequencies are shown as transconjugants per donor (*n* = 3). V200 possesses a higher conjugation frequency in all cases, revealing that EF3217 restricts DNA acquisition. The limit of detection was 100 CFU/ml. **, *P* < 0.01; ***, *P* < 0.001. Download FIG S3, TIF file, 0.8 MB. Copyright © 2018 Hullahalli et al. 2018 Hullahalli et al. This content is distributed under the terms of the Creative Commons Attribution 4.0 International license.

### Side effects of lethal chromosome targeting.

Since the genomes of E. faecalis clinical isolates typically possess multiple repetitive elements, we sought to assess whether CRISPR-mediated editing could select for large genome deletions or rearrangements. We used pGR-*ermB*, which targets *ermB* on pTEF1; pTEF1 is a 66-kb pheromone-responsive plasmid that naturally occurs in V583 and its derivatives and confers erythromycin and gentamicin resistance. Since *ermB* is flanked by two IS*1216* elements, we hypothesized that CRISPR targeting of *ermB* in the absence of an exogenous recombination template could result in erythromycin-sensitive mutants that had undergone recombination between the repetitive IS*1216* sequences. Indeed, multiple erythromycin-sensitive clones were recovered when *ermB* was targeted in strain V200. Whole-genome sequencing was performed on two of these mutants. In one clone (V202), the entire region between the IS*1216* transposases, including *ermB*, was deleted. Remarkably, the other clone (V204) had lost ~75% of pTEF1 (~45-kb deletion). V204 was also sensitive to gentamicin via deletion of *aac*(6′)-*aph*(2″). The mechanism for this large deletion was recombination between IS*1216* and IS*256* sequences on pTEF1 and pTEF3, which resulted in deletions in both plasmids (Fig. S4). Our findings demonstrate that CRISPR chromosome targeting can enrich for populations possessing larger recombination events in genomic regions where repetitive DNA is abundant, in agreement with previous data identifying large genomic rearrangements using CRISPR (45).

10.1128/mBio.00414-18.4 FIG S4  CRISPR targeting does not induce unintended single-nucleotide polymorphisms (SNPs) but drives large-scale recombination events. (A) Strain construction is shown. Red arrows indicate CRISPR editing, with the corresponding edits located adjacent to the arrows. The complete genomes of the underlined strains were sequenced. *pCE-*vanB* was not removed from V117 for this experiment. (B) Relevant mutations for the four sequenced strains are shown relative to the sequence of V117(pCE-*vanB*), since V117(pCE-*vanB*) possessed the fewest mutations. V200 (V583 P*_bacA_-cas9* ΔEF3217) and V204 (V583 P*_bacA_-cas9* ΔEF3217 Erm^s^ Gent^s^) differ from V117(pCE-*vanB*) by two SNPs, and V202 (V583 P*_bacA_-cas9* ΔEF3217 Erm^s^) differs from V117(pCE-*vanB*) by three SNPs. Mutations that were supposed to occur because of CRISPR editing and large-scale recombination events in the pTEF plasmids are not represented in this table but were confirmed by whole-genome sequencing. (C) Regions of deletion in pTEF1 and pTEF3 of V204 (V583 P*_bacA_-cas9* ΔEF3217 Erm^s^ Gent^s^) are shown as a red line. Relevant genes are indicated, and transposases that were found flanking the deleted region are underlined. Each graph represents the number of reads (from 0 to 2,000) as a function of the nucleotide position of each plasmid. The three lines at each position represent the minimum, mean, and maximum number of reads for each 1,000-nt or 100-nt grouping for pTEF1 and pTEF3, respectively. This grouping was automatically performed by CLC Genomics Workbench to display the data effectively when representing the entirety of the plasmid. Reads that mapped within the deleted regions were only those that mapped to multiple locations in the genome. V202 (V583 P*_bacA_-cas9* ΔEF3217 Erm^s^), which is not shown in this figure, contains a deletion of only *ermB*, mediated by recombination between the adjacent IS*1216* transposases. Download FIG S4, TIF file, 1 MB. Copyright © 2018 Hullahalli et al. 2018 Hullahalli et al. This content is distributed under the terms of the Creative Commons Attribution 4.0 International license.

Finally, we investigated potential off-target mutations that arose as a result of CRISPR-assisted genome editing, including whether unintended mutations occurred as a consequence of *cas9* overexpression. In addition to sequencing the genomes of strains V202 and V204 as described above, we sequenced V117(pCE-*vanB*) and V200 (see Fig. S4 for a diagram of strain derivations). These strains collectively represent three independent CRISPR-assisted editing events. V200 (V583 P*_bacA_-cas9* ΔEF3217) and V204 (V583 P*_bacA_-cas9* ΔEF3217 Erm^s^ Gent^s^) were identical (except for the aforementioned recombination events), while V117(pCE-*vanB*) and V202 (V583 P*_bacA_-cas9* ΔEF3217 Erm^s^) differed from V200 by 2 and 1 single-nucleotide polymorphisms, respectively (Fig. S4). The low frequency of genetic variations between the four clones confirms the highly specific nature of CRISPR-assisted genome editing in our system. This result is to be expected, given that editing is achieved by selecting for the desired recombinants, rather than promoting homology-directed repair. Taken together, our results validate CRISPR-assisted editing as a highly efficacious platform for genetic manipulation in E. faecalis.

## DISCUSSION

In this study, we investigated the intrinsic tolerance for chromosomal targeting by the native E. faecalis CRISPR1-*cas9*. We show that maintenance of chromosomal targeting constructs results in the induction of prophages but no induction of canonical SOS response genes, including *recA*. Furthermore, when *cas9* is overexpressed, a highly significant reduction in the number of transconjugants that accept CRISPR targeting constructs is observed. These transconjugants appear to be phenotypic CRISPR mutants. Using this knowledge, we subsequently developed a rapid and robust CRISPR-assisted genome-editing platform in E. faecalis.

We define CRISPR tolerance as the ability for CRISPR conflicts to be temporarily maintained, evidenced by reduced acquisition frequencies of targeted (or self-targeting) constructs, which precedes a selective pressure to relieve this conflict via mutation. Moreover, this transient nonlethality can be made lethal by modification (increased *cas9* expression). Variants of this phenotype have been observed in other organisms. In Pseudomonas aeruginosa, investigators found that an unusually large number of cells (2% relative to controls) were able to accept a CRISPR target and did not possess compensatory mutations in the protospacer/PAM (34). We infer that this CRISPR-Cas system may therefore also act in a tolerant manner. Similarly, in Listeria monocytogenes, a small-colony phenotype was observed following transformation of a CRISPR-targeted plasmid (46). Furthermore, tolerance of transcriptionally repressed targets has been directly demonstrated in a type III-A CRISPR-Cas system in Staphylococcus aureus, which only efficiently cleaves target DNA that is transcribed (47). This suggests that CRISPR-tolerant phenotypes occur in organisms other than E. faecalis.

We cannot be certain that low expression of *cas9* alone accounts for the ability of E. faecalis to survive chromosomal CRISPR targeting. It is possible that *trans*-acting factors, such as anti-CRISPR proteins encoded on mobile genetic elements (48), regulate the expression or activity of *cas9* in E. faecalis, thereby contributing to the CRISPR tolerance phenotype we observe. However, this particular scenario is unlikely because few mobile genetic elements are present in OG1RF and other non-MDR strains that display the CRISPR tolerance phenotype (24, 25). We have observed the CRISPR tolerance phenotype in five strains from five unique multilocus sequence types differing in genome size from 2.7 Mbp to 3.3 Mbp, suggesting that if an anti-CRISPR protein is involved, it is a component of the E. faecalis core genome (24, 25, 32). To this end, analysis of a prophage that is core to all E. faecalis strains (prophage 2) and a widely disseminated transposon (Tn*916*) using an anti-CRISPR database identified no genes with identity to known anti-CRISPR genes (49). Furthermore, anti-CRISPR genes have been shown to nullify the effect of CRISPR-Cas entirely, rather than generate the phenotype we observe in E. faecalis (46, 50). It nevertheless remains possible that *trans*-acting factors that have yet to be described in the literature contribute to CRISPR tolerance. Alternatively, *cas9* may be induced under certain conditions, which is probable given that we observe no antiphage activity of the native E. faecalis CRISPR-Cas under laboratory conditions, yet some E. faecalis CRISPR spacers are identical to phage sequences (9). The extent to which CRISPR tolerance occurs in the gastrointestinal tract and other environments where E. faecalis is found will be the subject of future investigations.

During preparation of the manuscript, a study by Jones et al. demonstrated that the kinetics of a catalytically inactive Cas9 are slow at low concentrations (51). The investigators suggest that in order for Cas9 to quickly find its target, both Cas9 and the crRNA would need to be present at high concentrations. It is therefore possible that the CRISPR tolerance we observe here and in our previous work is actually the direct phenotype of slow Cas9 kinetics in nature, and not due to inhibitory factors. This also implies that, at low concentrations, Cas9 is unable to efficiently cleave its target, and this leads to replication that proceeds faster than killing. The absence of DNA cleavage is also supported by our data, as we do not observe induction of DNA damage response genes, such as *recA*. However, if DNA cleavage is absent, the exact cause for prophage induction is unclear. It may be that the inefficient Cas9 is capable of killing some cells, which in turn release signals that promote prophage excision in neighboring cells, but this is highly speculative. Detecting the presence and number of DSBs in E. faecalis cells natively expressing *cas9* will further illuminate the mechanistic basis of CRISPR tolerance.

The advantage of CRISPR tolerance in the context of beneficial MGEs is clear. When CRISPR targets that may be beneficial are encountered by an E. faecalis population, it is advantageous for a large fraction of that population to be CRISPR tolerant and “sample” the effect of possessing the MGE. If the MGE is beneficial for survival, cells possessing it can still proliferate and CRISPR mutants emerge over time (25, 32); if the MGE is not beneficial, it can be lost or MGE-containing cells outcompeted. This may facilitate short-term acquisition of beneficial MGEs in commensal E. faecalis populations, while the absence of CRISPR-Cas activity may further predispose progenitors of high-risk MDR lineages to rapid genome expansion and adaptation to antibiotics.

## MATERIALS AND METHODS

### Bacterial strains, growth conditions, and routine molecular biology procedures.

Enterococcus faecalis was routinely cultured at 37°C in brain heart infusion (BHI) broth without agitation. Escherichia coli was routinely cultured at 37°C in lysogeny broth with agitation at 220 rpm. Routine PCR was performed with *Taq* DNA polymerase, and PCR for cloning purposes was performed with Q5 DNA polymerase (New England Biolabs). T4 polynucleotide kinase (New England Biolabs) was used for routine phosphorylation. PCR products were purified with the PureLink PCR purification kit (Invitrogen). Plasmids were purified using the GeneJet plasmid purification kit (Fisher). Primers were synthesized by Sigma-Aldrich. Routine DNA sequencing was performed at the Massachusetts General Hospital DNA Core Facility. E. coli EC1000 was used for routine plasmid propagation (52). E. faecalis and E. coli competent cells were prepared as described previously (25). Genomic DNA was extracted using the Mo Bio microbial DNA isolation kit (Qiagen). Antibiotics were used in the following concentrations: chloramphenicol, 15 µg/ml; streptomycin, 500 µg/ml; spectinomycin, 500 µg/ml; vancomycin (Van), 10 µg/ml; erythromycin (Erm), 50 µg/ml; rifampin, 50 µg/ml; fusidic acid, 25 µg/ml; tetracycline, 10 µg/ml; gentamicin (Gent), 300 µg/ml. A full list of primers can be found in Table S2 in the supplemental material.

10.1128/mBio.00414-18.8 TABLE S2  Primers used in this study. Download TABLE S2, DOCX file, 0.02 MB. Copyright © 2018 Hullahalli et al. 2018 Hullahalli et al. This content is distributed under the terms of the Creative Commons Attribution 4.0 International license.

### Strain and plasmid construction.

A schematic of the plasmid construction used in this study is shown in Fig. S5. All strains and plasmids used in this study are shown in Table S3. CRISPR-edited strains are shown in Table 1. All CRISPR-editing plasmids can be derived in a single step from pGR-*ermB* (accession number MF948287). The derivation of pGR-*ermB* is described below.

10.1128/mBio.00414-18.5 FIG S5  Plasmid construction scheme. The general plasmid workflow is shown (components not to scale). CRISPR repeats are depicted by thin, light-blue rectangles; the colored rectangles adjacent to the repeats represent various spacers. All CRISPR editing plasmids can be derived from pGR-*ermB* as either one-step or two-step assemblies. Generic primer schematic for generating CRISPR editing deletion plasmids from a single step is shown as arrows indicating 5′-to-3′ directionality. The primer pairs used in each reaction are colored identically (i.e., the two red arrows represent the primers that are used in the same reaction to amplify one fragment). Homologous overhangs for subsequent Gibson assembly are shown. Thirty-base-pair overhangs were used in all cloning procedures. Download FIG S5, TIF file, 0.6 MB. Copyright © 2018 Hullahalli et al. 2018 Hullahalli et al. This content is distributed under the terms of the Creative Commons Attribution 4.0 International license.

10.1128/mBio.00414-18.9 TABLE S3  Strains and plasmids used in this study. Download TABLE S3, DOCX file, 0.02 MB. Copyright © 2018 Hullahalli et al. 2018 Hullahalli et al. This content is distributed under the terms of the Creative Commons Attribution 4.0 International license.

To generate chromosome-targeting constructs, pCR2*-ermB* was linearized to remove 160 bp upstream from the *ermB* spacer and simultaneously introduce the promoter of *bacA* from pPD1, which is constitutive (P_*bacA*_) (25, 35). This procedure also removed the upstream repeat. The linear product was phosphorylated and self-ligated to generate an intermediate plasmid referred to as pSR-*ermB*. This plasmid was once again linearized around *cat*, and a fragment containing *cat* and pheS* from pLT06 was blunt end ligated (53). The original *cat* was deleted to simplify the cloning procedure. The final plasmid was designated pGR-*ermB* and was fully sequenced (accession number MF948287).

To modify the spacer, pGR-*ermB* was linearized at P_*bacA*_ and the downstream repeat; primers contained the entirety of the spacer sequence to be inserted. The exception was pGR-IS*256*, which was generated without ligation by taking advantage of the ability of E. coli EC1000 to recombine linear DNA (i.e., linear DNA was recombined *in vivo*). All pGR derivatives were sequence verified to ensure spacer integrity prior to introduction into strain C173 for conjugation. Homologous recombination templates were introduced using the NEB HiFi DNA assembly master mixture (New England Biolabs). For simplicity, the spacer was included as overhangs during Gibson assembly, and therefore, a plasmid containing two fragments for homologous recombination and the appropriate spacer could be generated in a single step. The same linearization-phosphorylation-ligation procedure was used to modify the plasmid to insert P_*bacA*_ upstream from *cas9*. Knock-in protocols were performed essentially as previously described (54). A streamlined protocol for CRISPR-assisted genome editing in E. faecalis using our system is outlined in Fig. S6, and the primer schematic for generating CRISPR editing plasmids is shown in Fig. S5.

10.1128/mBio.00414-18.6 FIG S6  CRISPR-Cas genome-editing protocol for E. faecalis. A workflow for achieving CRISPR-assisted genome editing in E. faecalis is shown. (1) Overnight cultures of donors (D) and recipients (R) are grown. Donors must possess the appropriate CRISPR editing plasmid, and recipients must possess a sufficiently expressed *cas9*. (2) Overnight cultures are diluted in BHI without antibiotics, regrown for 1.5 h, and (3) subsequently plated at a donor-to-recipient ratio of 1:9 on BHI. (4) The conjugation reaction mixture is incubated overnight and then scraped and plated on appropriate selective medium to obtain transconjugants (TCs), which are then (5) restruck on medium containing chloramphenicol. (6) Colonies are then inoculated into BHI broth, cultured until turbid, and (7) plated on MM9YEG plus *p*-Cl-Phe to counterselect for the plasmid. Edited clones are subsequently confirmed to be chloramphenicol sensitive. Media are color coded. BHI, BHI plus chloramphenicol, and MM9YEG plus *p*-Cl-Phe are shown in red, brown, and green, respectively. The bacteria present at each step of the process are also indicated. The appropriate number of colonies to screen from the initial transconjugant selection is dependent on each experiment, but we find that proceeding with 6 unique transconjugants is sufficient to recover at least two edited clones. Download FIG S6, TIF file, 0.8 MB. Copyright © 2018 Hullahalli et al. 2018 Hullahalli et al. This content is distributed under the terms of the Creative Commons Attribution 4.0 International license.

For CRISPR-assisted editing, the appropriate plasmid was first transformed into E. faecalis C173 or CK111SSp(pCF10-101). Conjugation into the desired recipient strain was then performed, and transconjugants were selected on agar medium containing chloramphenicol and appropriate antibiotics for recipient strain selection. Transconjugant colonies were restruck for isolation on agar medium containing chloramphenicol, and single colonies were inoculated into 1 to 5 ml of BHI broth lacking antibiotics and incubated at 37°C until turbid. Cultures were then struck on MM9YEG (36) containing *para*-chloro-phenylalanine (*p*-Cl-Phe) to counterselect against the plasmid backbone. By this point, the recipient strain will have received the CRISPR editing plasmid, recombined with the editing template, and then lost the backbone plasmid. In total, this procedure can take as little as 2 days once transconjugants are obtained. We observed that an additional passage in MM9YEG–*p*-Cl-Phe was helpful for eliminating residual chloramphenicol resistance, since the counterselection is imperfect. This extra passage was utilized whenever frequencies needed to be determined and there was no marker to phenotypically screen for, since preliminary experiments occasionally yielded some chloramphenicol-resistant clones which interfered with an accurate assessment of successful editing rates. Once presumptive CRISPR-edited mutants were obtained, colony PCR to confirm the desired edit was performed in all cases except for deletion of *pstB*; the larger amplicon required that genomic DNA be extracted. Genotypes of representative clones were verified through Sanger sequencing (for deletion of *pstB*, *pstSCAB*, and *vanB*) or whole-genome sequencing (for deletion of EF3217).

### Conjugation assays.

Conjugation assays were performed essentially as described previously (25). C173 was used as the donor in all experiments, except for experiments using CRISPR-mediated editing to delete *vanB*. For deletion of *vanB*, the erythromycin-sensitive strain CK111SSp(pCF10-101) was used as the donor, since transconjugant selection during this experiment required erythromycin instead of vancomycin and C173 is erythromycin resistant.

### Transcriptomics analysis.

To assess the transcriptional response to CRISPR self-targeting, transconjugants of V649(pGR-*tetM*) (control) and V649(pGR-IS*256*) (test) selected on vancomycin and chloramphenicol were incubated on agar medium for 2 days. Cells were scraped from plates, resuspended in RNA-Bee (Tel-Test, Inc.), and lysed by bead beating in lysis matrix B (MP Biomedicals). After RNA-Bee extraction, the aqueous layer was subjected to ethanol precipitation. The RNA was treated with DNase (Roche) and concentrated using the GeneJet RNA cleanup and concentration kit (Fisher). For assessment of the transcriptional response to levofloxacin (LVX)-induced stress, cells were treated essentially as previously described (25). Briefly, overnight cultures of V649 were diluted in fresh medium and grown to an optical density at 600 nm (OD_600_) of 0.3, at which point cultures were split. Some cells were harvested for control transcriptomic analysis, and LVX was added to the remaining cells at a concentration of 1 µg/ml. After 2 h of incubation with LVX, the remaining cells were harvested. RNA was isolated and treated with DNase as described above. Three biological replicates were performed under both experimental conditions.

RNA-Seq analysis was performed at MR DNA (Molecular Research LP). The concentration of total RNA was determined using the Qubit RNA assay kit (Life Technologies, Inc.). Baseline-Zero DNase (Epicentre) was used to remove DNA contamination, and the RNA was purified using RNA Clean and Concentrator-5 columns (Zymo Research). Subsequently, rRNA was removed by using the Ribo-Zero gold rRNA removal (epidemiology) kit (Illumina) and purified with RNA Clean and Concentrator-5 columns (Zymo Research). rRNA-depleted samples were subjected to library preparation using the TruSeq RNA LT sample preparation kit (Illumina) according to the manufacturer’s instructions. The libraries were pooled and paired-end sequenced for 300 cycles using the HiSeq 2500 system (Illumina).

RNA-sequencing data were analyzed using CLC Genomics Workbench. rRNA and tRNA reads were first removed, and the unmapped reads were mapped to the V649 reference genome. Transcripts-per-million (TPM) values were used to quantitate expression. False discovery rate (FDR)-adjusted *P* values were used to assess significance. Genes were filtered by first removing those for which both CRISPR self-targeting and LVX treatment yielded FDR-adjusted *P* values of >0.05. Subsequently, genes for which both LVX treatment and CRISPR self-targeting had fold changes of <2 were removed. The remaining list consisted of genes that were significantly up- or downregulated by either LVX treatment or CRISPR self-targeting. Raw reads for RNA sequencing and whole-genome sequencing have been deposited in the Sequence Read Archive under accession number PRJNA420898.

RT-qPCR to verify increased *cas9* expression was performed as previously described (25). RNA was harvested from cultures of strains V649 and V117 at an OD_600_ of 0.3.

### Phage resistance assay.

Approximately 10^5^ to 10^6^ PFU/ml of ΦNPV-1 was added to 5 ml of M17 soft agar (Fisher) plus chloramphenicol and overlaid on BHI agar plus chloramphenicol (41). Overnight cultures of strains OG1RF and OG117 containing pGR-*tetM* or pGR-NPV1 were spotted on the soft agar containing ΦNPV1. pGR-NPV1 targets a predicted phage lysin gene. A simultaneous control lacking soft agar and phage was included to enumerate total bacterial CFU. Using identical amounts of ΦNPV-1 in each experiment was essential for consistent results.

### Detection of circular Phage01 DNA.

Cultures were treated identically to those prepared for RNA sequencing. Cells were pelleted, and genomic DNA was extracted using the Mo Bio microbial DNA isolation kit (Qiagen) according to the manufacturer’s instructions. qPCR was performed using the AzuraQuant green fast qPCR mixture LoRox (Azura) according to the manufacturer’s instructions. Similar to a previously reported approach for circular-phage detection (39), circular Phage01 DNA was detected using primers qpp1c For and qpp1c Rev, which amplify across the junction of the circularized phage.

### Phage lysis assay.

Cultures were induced with LVX as described in a previous section. Induced cultures were pelleted, and the supernatant was filtered using 0.2-µm polyethersulfone filters. Similarly, transconjugant colonies of V649(pGR-*tetM*) and V649(pGR-IS*256*) were scraped from agar plates using 2 ml phosphate-buffered saline (PBS) (identical to the protocol used for transcriptomics analysis) and pelleted, and the supernatant filtered. Filtrates were spotted on soft agar containing lawns of E. faecalis ATCC 29212, which is susceptible to infection by V583 prophages (55). To prepare the lawns, overnight cultures of ATCC 29212 were diluted in fresh medium and cultured to an OD_600_ of 0.4. Amounts of 10 μl of culture were added to 2-ml amounts of melted soft agar (BHI broth, 0.2% agarose, 10 mM MgSO_4_), and the mixtures were poured onto 100-mm-diameter standard BHI agar plates (1.5% agar). We observed that varying the amount of bacteria added and the thickness of the soft agar affected the visibility of phage plaques; the protocol we present here yielded the clearest zones of lysis.

### Genome sequencing.

Whole-genome sequencing was performed at MR DNA (Molecular Research LP). Briefly, libraries were prepared with the Nextera DNA sample preparation kit (Illumina) using 50 ng of total genomic DNA. Libraries were pooled and paired-end sequenced for 300 cycles using the Illumina HiSeq system. Reads were mapped to the V117 genome in CLC Genomics Workbench. Mapping graphs were generated to identify deleted (zero-coverage) regions, and basic variant detection was performed on read mappings to identify smaller single-nucleotide polymorphisms (SNPs), deletions, and insertions using the default parameters. Raw reads for RNA sequencing and whole-genome sequencing have been deposited in the Sequence Read Archive under accession number PRJNA420898.

### Statistics.

*P* values for conjugation frequencies and CFU measurements were calculated using a one-tailed Student’s *t* test from log_10_-transformed values. *P* values for qPCR data were calculated using a one tailed Student’s *t* test. Geometric mean values and geometric standard deviations are shown for all data except those presented in Table 1 (CRISPR editing experiments).

### Accession number(s).

The pGR-*ermB* sequence has been deposited in GenBank under accession number MF948287. Raw reads for RNA sequencing and whole-genome sequencing have been deposited in the Sequence Read Archive under accession number PRJNA420898.
