# Erratum for Hullahalli et al., “An Attenuated CRISPR-Cas System in Enterococcus faecalis Permits DNA Acquisition”

**DOI:** 10.1128/mBio.01775-19

**Published:** 2019-08-13

**Authors:** Karthik Hullahalli, Marinelle Rodrigues, Uyen Thy Nguyen, Kelli Palmer

**Affiliations:** a Department of Biological Sciences, The University of Texas at Dallas, Richardson, Texas, USA

## ERRATUM

Volume 9, issue 3, e00414-18, 2018, https://doi.org/10.1128/mBio.00414-18. Plasmid maps show some components in the incorrect location and orientation. This does not affect any of the results of the study, and the plasmid sequence uploaded to GenBank (pGR-ermB; accession number MF948287) possesses correctly annotated components. Figure 1 and Fig. 6 (corrected below) and Fig. S2, S5, and S6 in the supplemental material (replaced online) were affected and have been updated to reflect the correct locations and orientations of pheS*, cat, and the CRISPR guide sequence.

**FIG 1:**
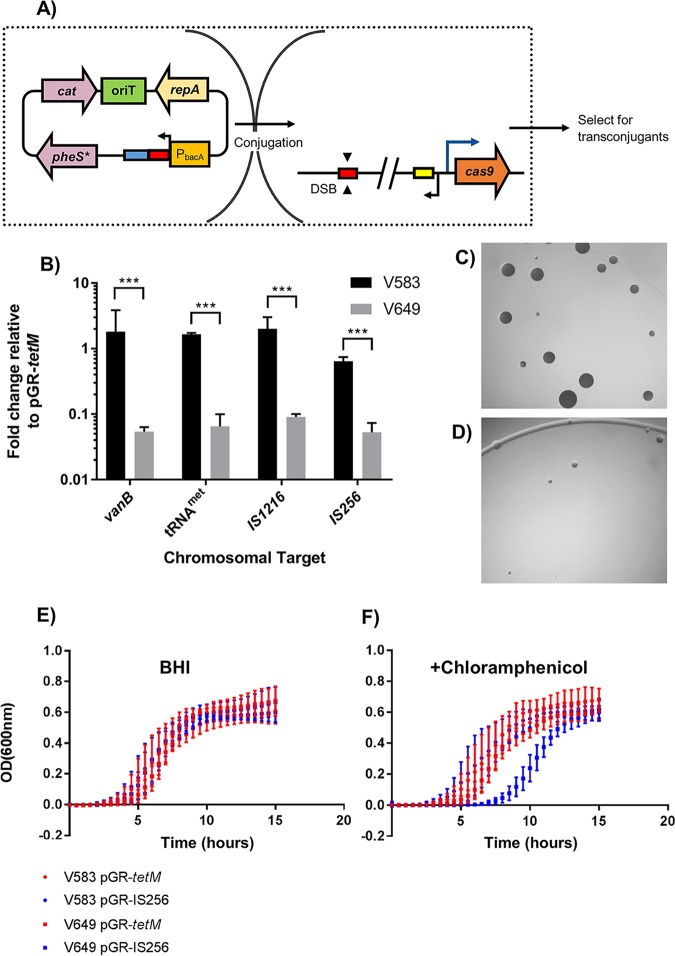
CRISPR tolerance protects against self-targeting.

**FIG 6:**
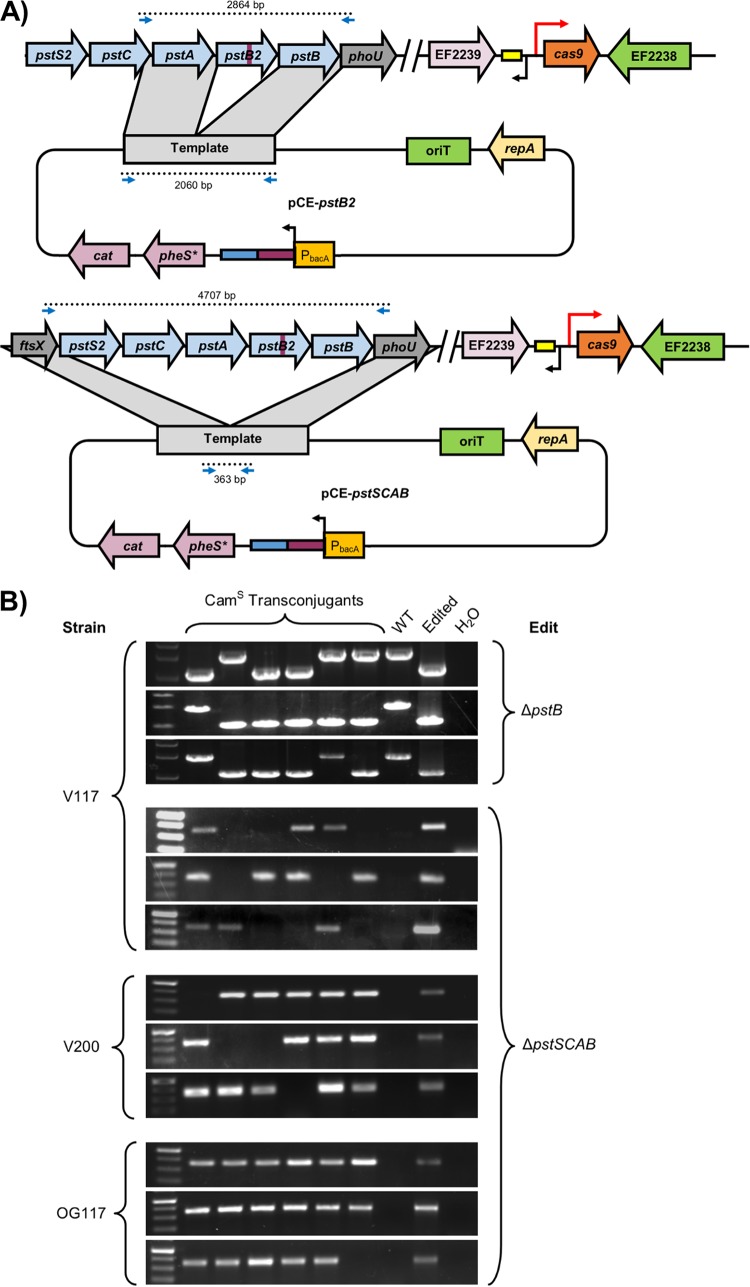
CRISPR editing in E. faecalis.

