# Supplementary figures and images for: An Attenuated CRISPR-Cas System in Enterococcus faecalis Permits DNA Acquisition
Source: mBio. 2018 May 1;9(3):e00414-18. doi: 10.1128/mBio.00414-18 (PMC5930301; doi:10.1128/mBio.00414-18)

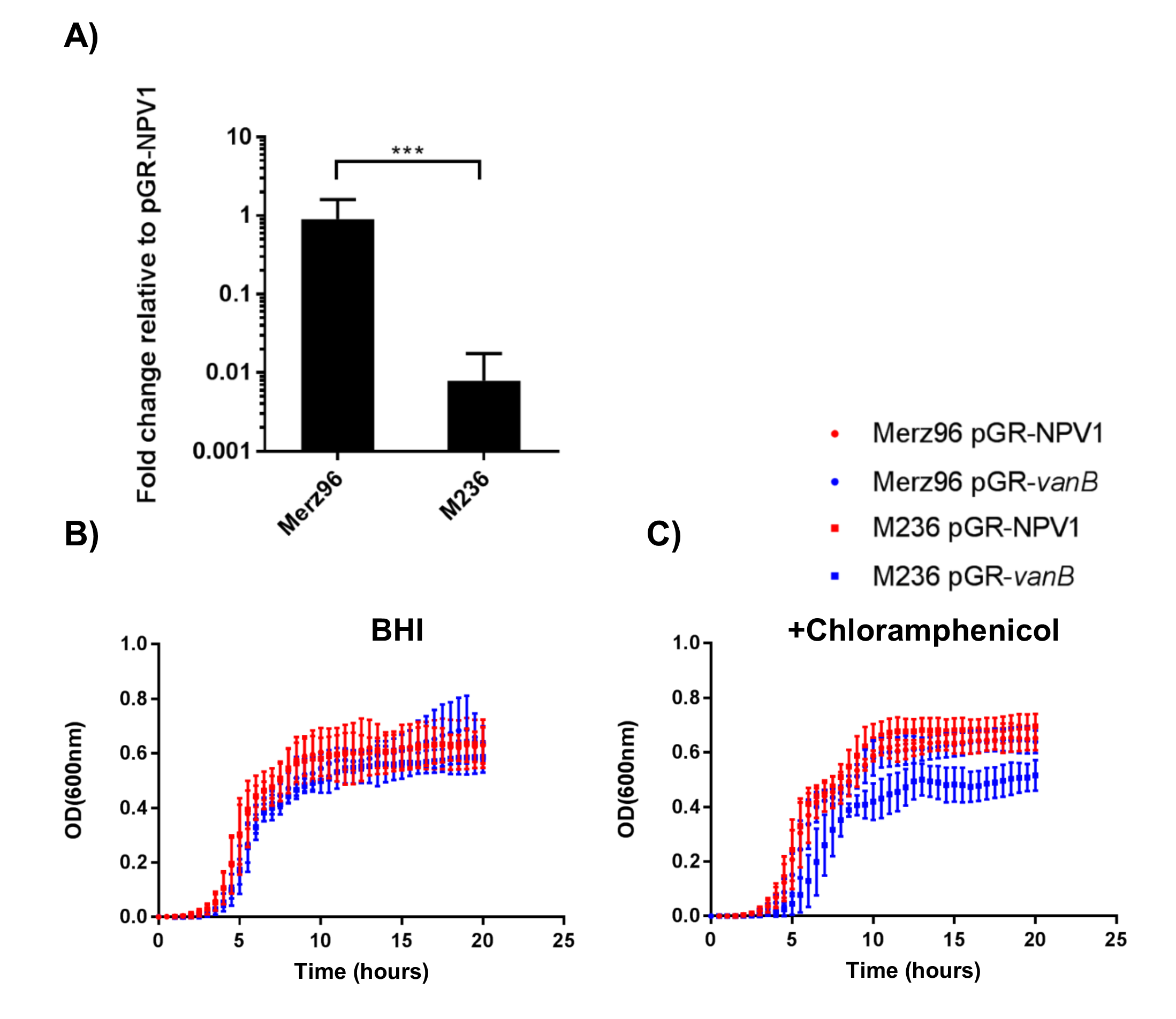

Supplement: FIG S1 [file mbo002183850sf1.tif]

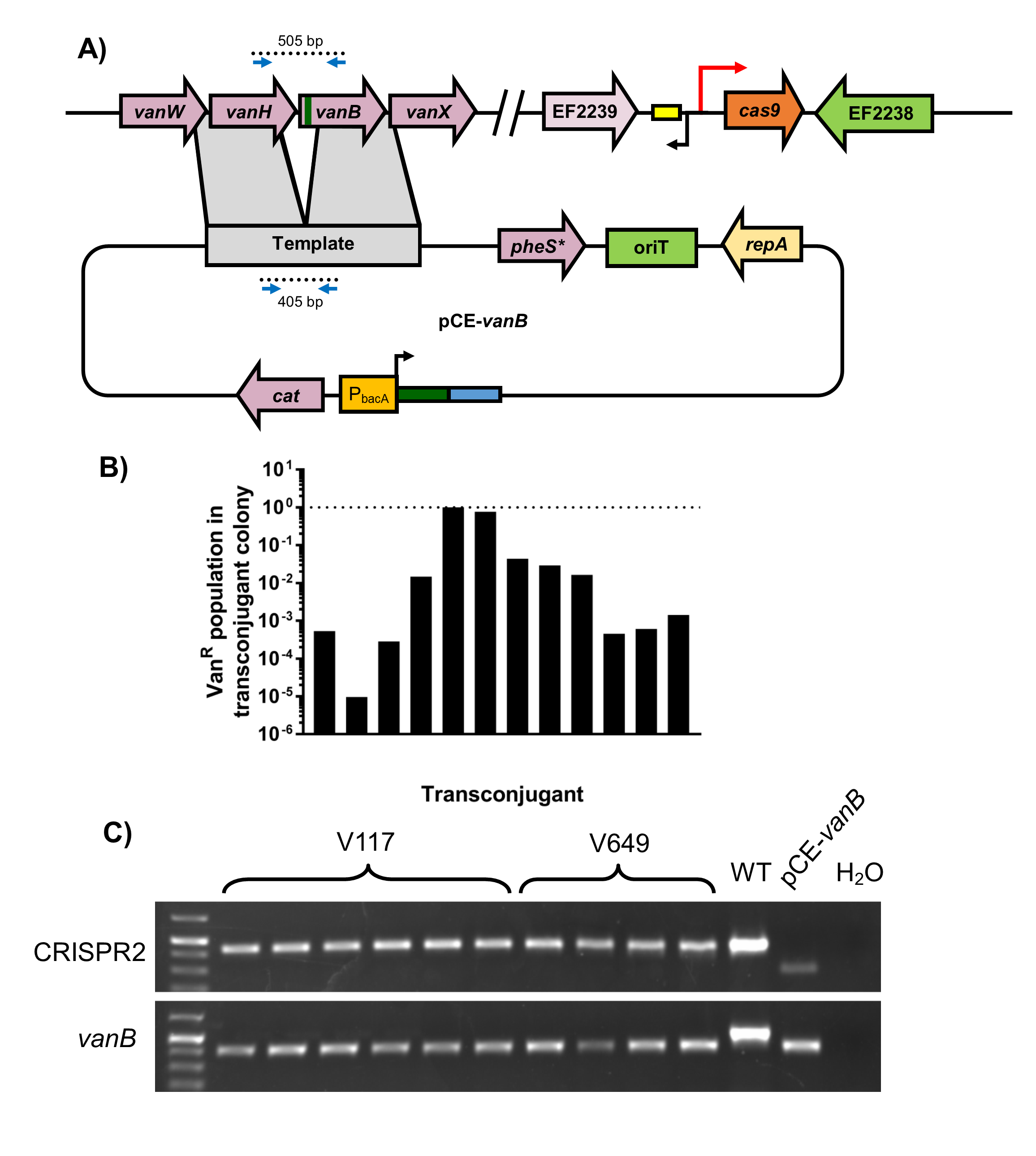

Supplement: FIG S2 [file mbo002183850sf2.tif]

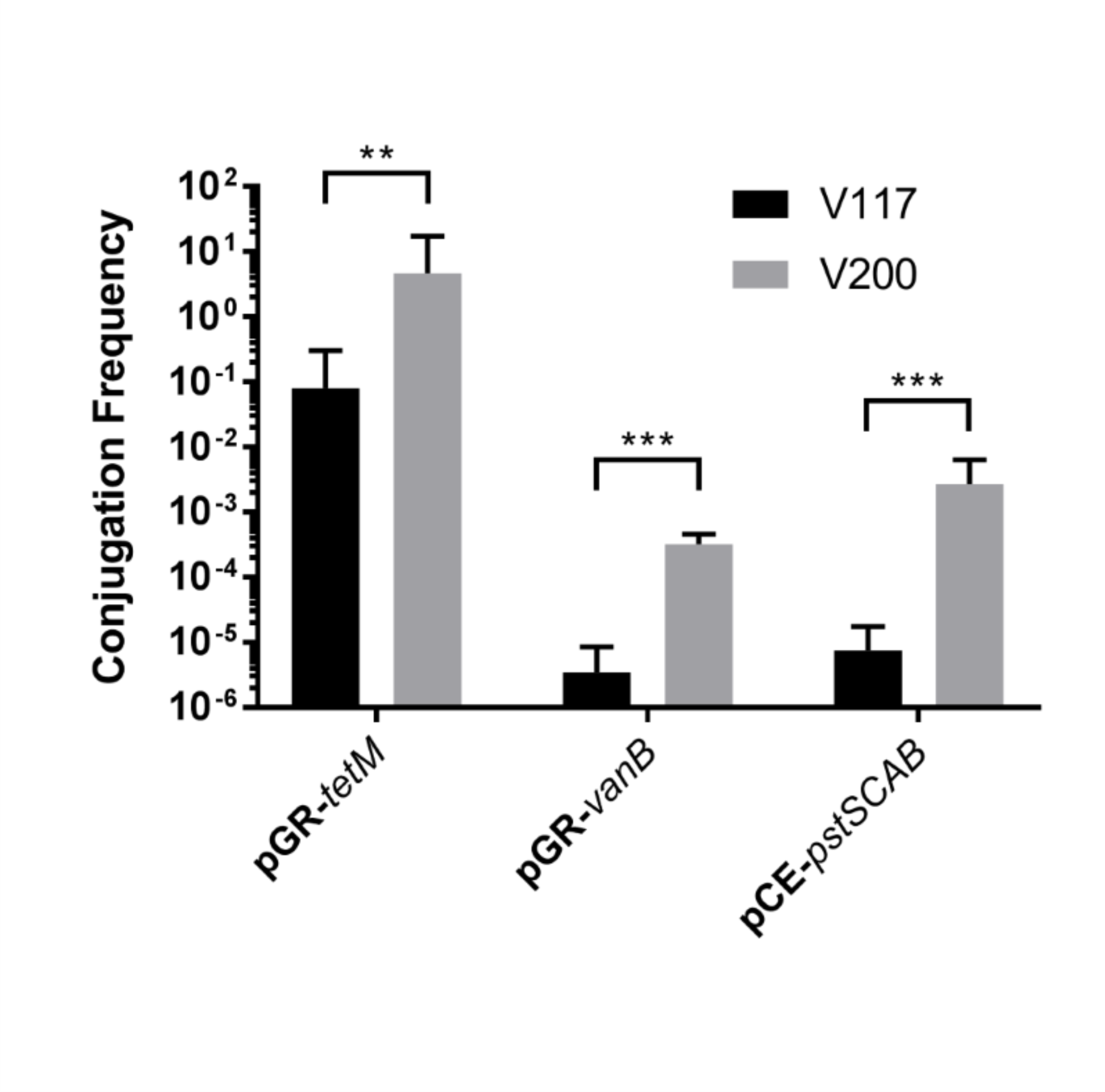

Supplement: FIG S3 [file mbo002183850sf3.tif]

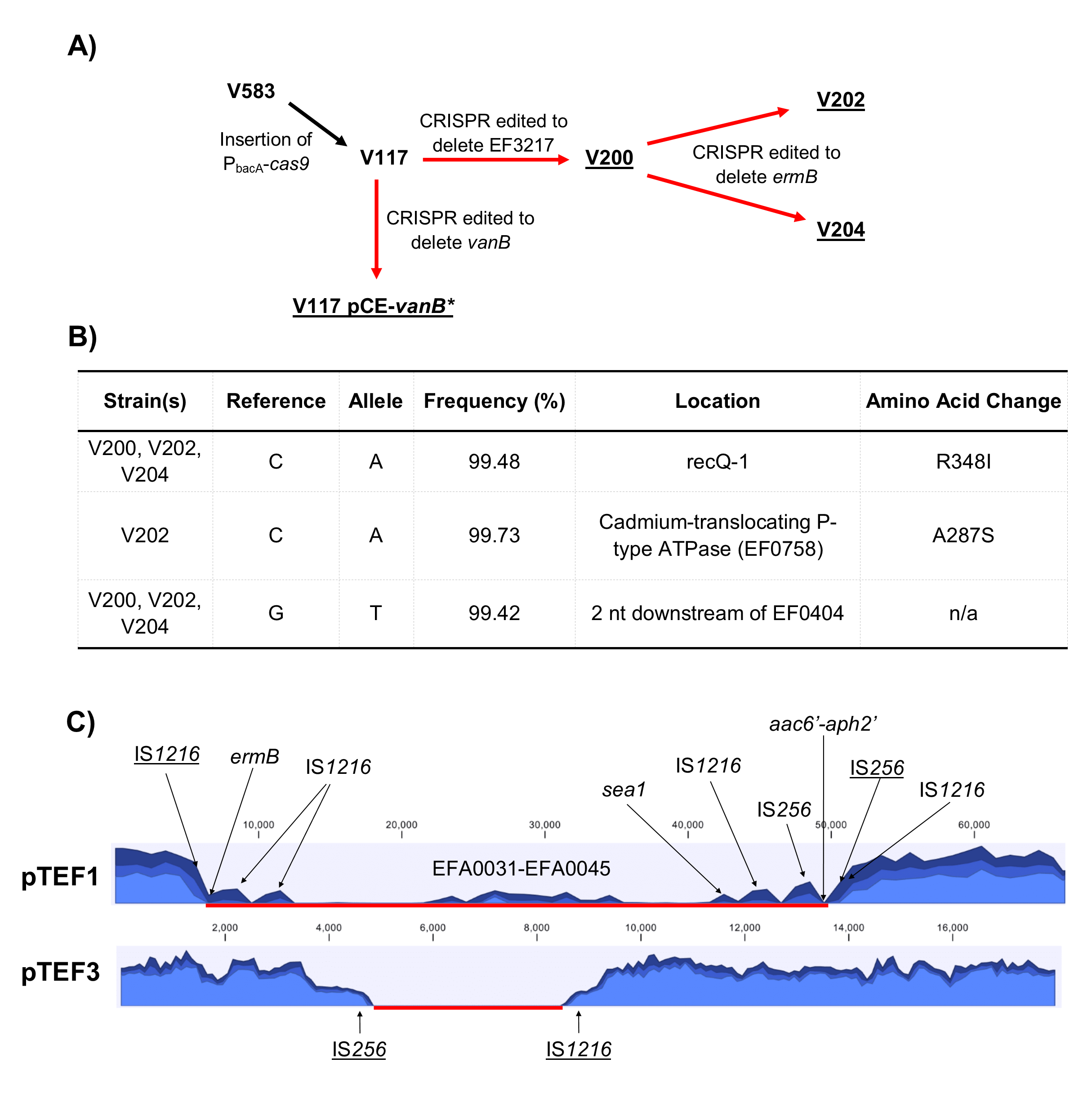

Supplement: FIG S4 [file mbo002183850sf4.tif]

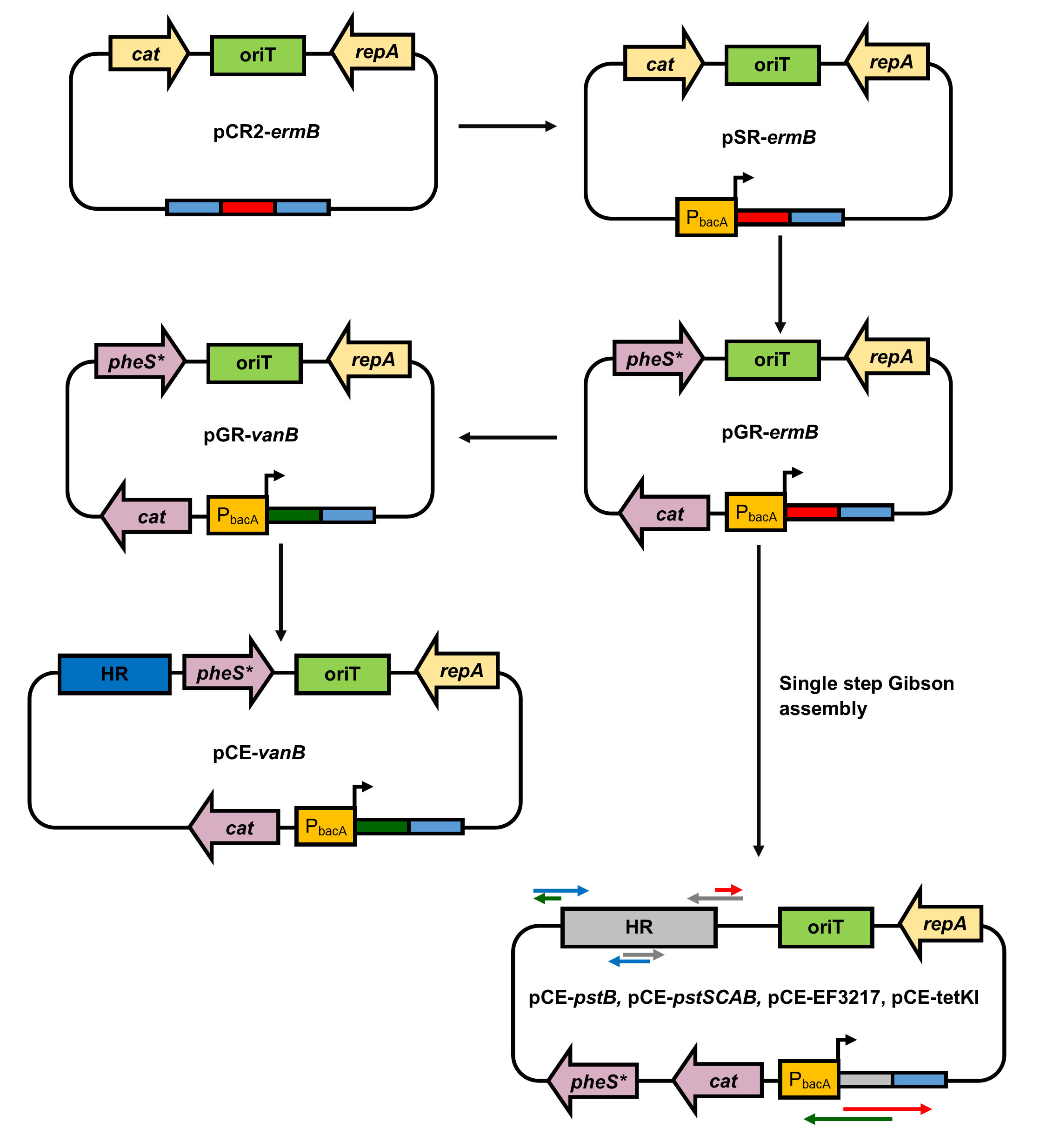

Supplement: FIG S5 [file mbo002183850sf5.tif]

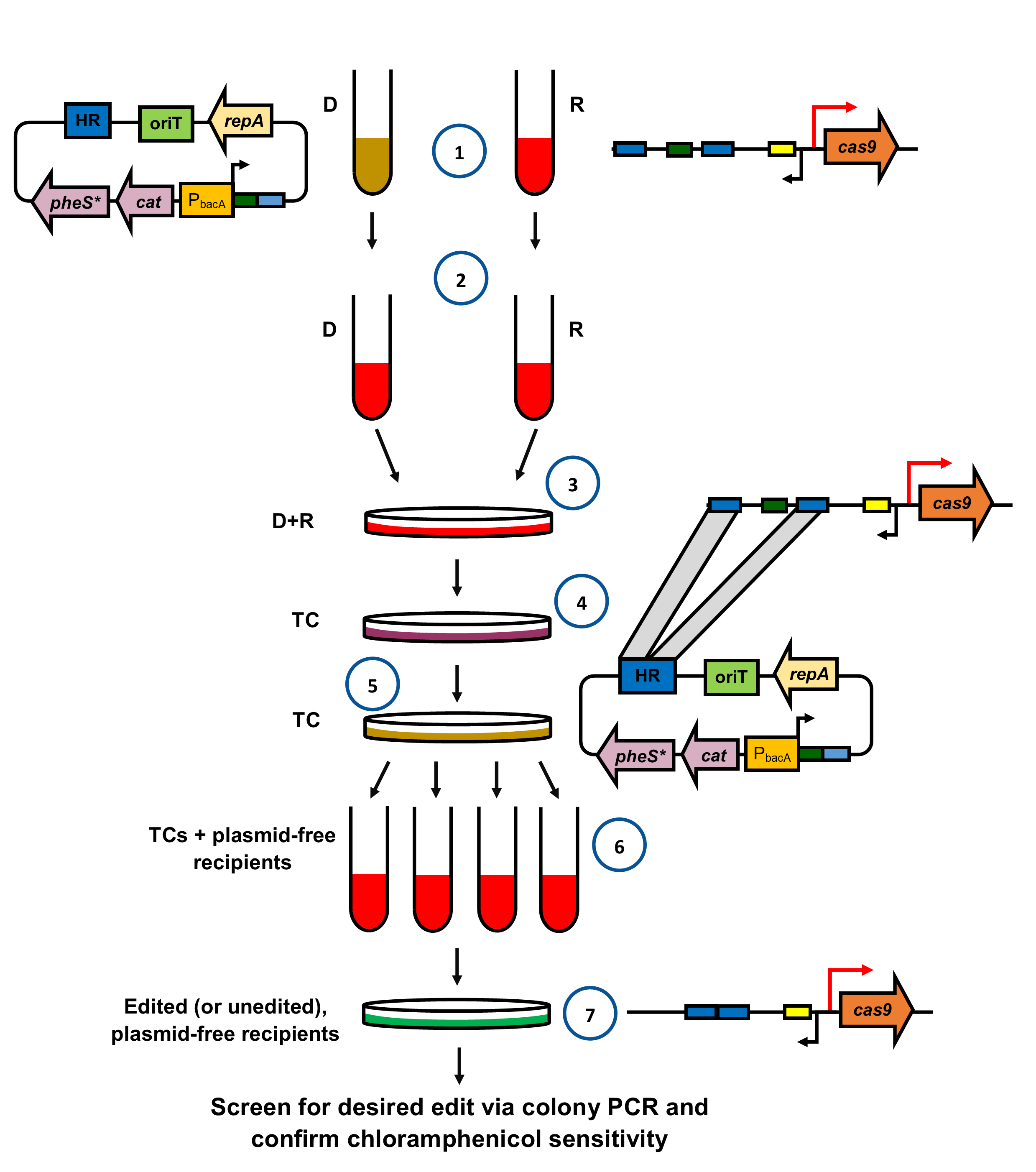

Supplement: FIG S6 [file mbo002183850sf6.tif]
