# Supplementary material for: An Attenuated CRISPR-Cas System in Enterococcus faecalis Permits DNA Acquisition
Source: mBio. 2018 May 1;9(3):e00414-18. doi: 10.1128/mBio.00414-18 (PMC5930301; doi:10.1128/mBio.00414-18)
Supplement: TABLE S1 [file mbo002183850st1.docx]

|  | V583 pGR-tetM | V583 pGR-IS256 | V649 pGR-tetM | V649 pGR-IS256 |
| --- | --- | --- | --- | --- |
| Levofloxacin | 1 | 1 | 1 | 0.5 |
| Ciprofloxacin | 1 | 1 | 1 | 0.5 |
