# Supplementary material for: An Attenuated CRISPR-Cas System in Enterococcus faecalis Permits DNA Acquisition
Source: mBio. 2018 May 1;9(3):e00414-18. doi: 10.1128/mBio.00414-18 (PMC5930301; doi:10.1128/mBio.00414-18)
Supplement: TABLE S2 [file mbo002183850st2.docx]

| **Primer Name** | **Sequence (5'-3')** | **Use** |
| --- | --- | --- |
| PbacA CR2 lin rev | TTTATTTTGATGCAAGCAATAACATAAAAACCCACCATTTTTCAATGG | Insert PbacA into pCR2-*ermB* |
| PbacA erm lin for | CTACATGGGTATAATAGCAATGAAATTGTTGAAGAAGGATTCTACAAGCG | Insert PbacA into pCR2-*ermB* |
| Tet lin fev | CGAACTTTACCGAATCTGAACAATGGGATAGTTTTAGAGTCATGTTGTTTAG | Create pGR-*tetM* |
| Tet lin for | TTTCATTGCTATTATACCCATG | Create pGR-*tetM* |
| Van lin for | AGGAACATGATGTGTGTTTTAGAGTCATGTTGTTTAG | Create pGR-*vanB* |
| Van lin rev | CCGAGCAACCGCCGATTTCATTGCTATTATACCCATG | Create pGR-*vanB* |
| Met lin for | GCTGGTTAGAGCAAAGTTTTAGAGTCATGTTGTTTAG | Create pGR-met |
| Met lin rev | TGAGCTAATGGTCCATTTCATTGCTATTATACCCATG | Create pGR-met |
| 1216 lin for | TAGAATTTATTGCGTCTCTTTACTGGACGAGTTTTAGAGTCATGTTGTTTAG | Create pGR-IS1216 |
| 1216 lin rev | TTTCATTGCTATTATACCCATG | Create pGR-IS1216 |
| 256 lin for | AAAAATGGCCATCACGTGTTCGTTTTAGAGTCATGTTGTTTAG | Create pGR-IS256 |
| 256 lin rev | ATGGCCATTTTTCACCCACAGTTTCATTGCTATTATACCCATG | Create pGR-IS256 |
| NPV1 Lin For | ATACGGTCACACAGGAATTGCAACGGAGGAGTTTTAGAGTCATGTTGTTTAG | Create pGR-NPV1 |
| NPV1 Lin Rev | TTTCATTGCTATTATACCCATG | Create pGR-NPV1 |
| PbacA Cas9 for | CTACATGGGTATAATAGCAATGAAATAGTAATTTAAAAAAAGGAGTGG | Insert PbacA into the *cas9* promoter |
| PbacA Cas9 rev | TTTATTTTGATGCAAGCAATAACATCTTGAATGATTTTTATTCTATGC | Insert PbacA into the *cas9* promoter |
| Spe pheS for | GAGGATGAGGAGGCAGATTGC | Create pCE-*vanB* |
| pLZ12 MCS rev | TCCACTCCTGAATCCCATTCC | Create pCE-vanB and pGR-*ermB* |
| pCE-vanB Arm1 for | AGAATTTCTGGAATGGGATTCAGGAGTGGAGCGAACCAATGAGAAAAAGTATG | Create pCE-*vanB* |
| pCE-vanB Arm1 rev | GCGGATCGAATTTTTGCTGTAAACTCCTTTCAAAGTTAAG | Create pCE-*vanB* |
| pCE-vanB Arm2 for | AAAGGAGTTTACAGCAAAAATTCGATCCGCACTAC | Create pCE-*vanB* |
| pCE-vanB Arm2 fev | ATATTCAAGGCAATCTGCCTCCTCATCCTCTAAAAACAAAAAACCATTTTCCATAC | Create pCE-*vanB* |
| del vanB screen for | ATCATCACACCCCATACGGC | Screen for *vanB* edit |
| del vanB screen rev | GGCCAGTGATTTGTCCATGC | Screen for *vanB* edit |
| pCE ori for | TTTCTGAACCGACTTCTCCTTTTTC | Create pCE-pstB and pCE-pstSCAB |
| pCE pheS cat rev | AAGAAGGATATGGATCTGGAG | Create pCE-pstB and pCE-pstSCAB |
| pCE-pstB pheS cat for | TAACTTAAACAAAAGCGCCTTAGCTCTGTCGTTTTAGAGTCATGTTGTTTAGAATGG | Create pCE-pstB, to delete *pstB* |
| pCE-pstB ori rev | GACAGAGCTAAGGCGCTTTTGTTTAAGTTATTTCATTGCTATTATACCCATGTAG | Create pCE-pstB, to delete *pstB* |
| pCE-pstB Arm1 for | ATATTACAGCTCCAGATCCATATCCTTCTTCAACGTTCTTTGGTCTTTAGCC | Create pCE-pstB, to delete *pstB* |
| pCE-pstB Arm1 rev | ATCTTGCTCCTCCTACATGCTAATTCCCCTAACATTAAGC | Create pCE-pstB, to delete *pstB* |
| pCE-pstB Arm2 for | AGGGGAATTAGCATGTAGGAGGAGCAAGATGGGC | Create pCE-pstB, to delete *pstB* |
| pCE-pstB Arm2 rev | GAAGCGAAAAAGGAGAAGTCGGTTCAGAAAGTTGTAACGCAATCATTTCAAAACTC | Create pCE-pstB, to delete *pstB* |
| pCE-pstSCAB pheS cat for | TAACTTAAACAAAAGCGCCTTAGCTCTGTCGTTTTAGAGTCATGTTGTTTAGAATGG | Create pCE-pstSCAB, to delete *pstB* |
| pCE-pstSCAB ori rev | GACAGAGCTAAGGCGCTTTTGTTTAAGTTATTTCATTGCTATTATACCCATGTAG | Create pCE-pstSCAB, to delete *pstB* |
| pCE-pstSCAB Arm1 for | ATATTACAGCTCCAGATCCATATCCTTCTTATGACTGTTGCCTCAGCAAG | Create pCE-pstSCAB, to delete *pstB* |
| pCE-pstSCAB Arm1 rev | AGAAATGTAATCTTCCATCGATTCATTATTCCTCCAATT | Create pCE-pstSCAB, to delete *pstB* |
| pCE-pstSCAB Arm2 for | AATAATGAATCGATGGAAGATTACATTTCTGGTAAATTTGG | Create pCE-pstSCAB, to delete *pstB* |
| pCE-pstSCAB Arm2 rev | GAAGCGAAAAAGGAGAAGTCGGTTCAGAAATTTTTCAGTTGCCATATTTTCTAATA | Create pCE-pstSCAB, to delete *pstB* |
| pCE-pstSCAB screen for | AGGTTCAGTTATTTCAATGCGTCG | Screen for pstSCAB edit |
| pCE-pstSCAB screen rev | GCCTTCACGGATTTATGGACGGC | Screen for pstSCAB edit |
| pKH12 cat lin for | CATGAGATAATGCCGACTGTAC | Create pGR-*ermB* |
| pCE-3217 ori Rev | GATAAATAAGCACTCGGAATTCCACGATCGTTTCATTGCTATTATACCCATGTAG | Create pCE-3217, to delete EF3217 |
| pCE-3217 pheS Cat For | CGATCGTGGAATTCCGAGTGCTTATTTATCGTTTTAGAGTCATGTTGTTTAGAATGG | Create pCE-3217, to delete EF3217 |
| pCE-3217 Arm1 For | ATATTACAGCTCCAGATCCATATCCTTCTTGCTCGTAAAGCTTCACAGTTCTC | Create pCE-3217, to delete EF3217 |
| pCE-3217 Arm1 Rev | AAAGTGGCTTTTTTATTCTAAATTATCCATTTTGTTCAGTTCCC | Create pCE-3217, to delete EF3217 |
| pCE-3217 Arm2 For | ATGGATAATTTAGAATAAAAAAGCCACTTTCCTCTGG | Create pCE-3217, to delete EF3217 |
| pCE-3217 Arm2 Rev | GAAGCGAAAAAGGAGAAGTCGGTTCAGAAATAAAAGTTTGAAACCGCAAATTC | Create pCE-3217, to delete EF3217 |
| pCE-tetKI ori Rev | ACATCATTTGACAAAGAGCCTTTATACTACTTTCATTGCTATTATACCCATGTAG | Create pCE-tetKI, to knock-in *tetM* |
| pCE-tetKI pheS Cat For | GTAGTATAAAGGCTCTTTGTCAAATGATGTGTTTTAGAGTCATGTTGTTTAGAATGG | Create pCE-tetKI, to knock-in *tetM* |
| pCE-tetKI Arm1 For | ATATTACAGCTCCAGATCCATATCCTTCTTAAGAAACAAAATTTGTATCAGAAGC | Create pCE-tetKI, to knock-in *tetM* |
| pCE-tetKI Arm1 Rev | CCGTTCTTTTCAAGTACTCTCATTTTTGGTGCTAAAAAG | Create pCE-tetKI, to knock-in *tetM* |
| pCE-tetKI tetM For | ACCAAAAATGAGAGTACTTGAAAAGAACGGGAGTAATTGG | Create pCE-tetKI, to knock-in *tetM* |
| pCE-tetKI tetM Rev | TAAGATTTCTCTTTATTCCACATACAGGACACAATATCC | Create pCE-tetKI, to knock-in *tetM* |
| pCE-tetKI Arm2 For | GTCCTGTATGTGGAATAAAGAGAAATCTTAGAATAATTTGGAC | Create pCE-tetKI, to knock-in *tetM* |
| pCE-tetKI Arm2 Rev | GAAGCGAAAAAGGAGAAGTCGGTTCAGAAAATTGCTCGCTTAAAAGAGAATAC | Create pCE-tetKI, to knock-in *tetM* |
| pCE-tetKI Screen For | GGTGGGGCAGAAGCTGAAGG | Create pCE-tetKI, to knock-in *tetM* |
| pCE-tetKI Screen Rev | ACCTTGCCGCATATTTATTAACTCC | Create pCE-tetKI, to knock-in *tetM* |
| qpp1C For | TTGCCCTTTTTGTGCCCTTTTCC | qPCR for circular phage01 |
| qpp1C Rev | TTTTTGTGAAAATTGGACCAAATCCTTGGG | qPCR for circular phage01 |
| qvanB For | AAGCCGATAGTCTCCCCGCC | qPCR for *vanB* |
| qvanB Rev | CCATCCTCCCCGCATTTGCC | qPCR for *vanB* |
| qcas9 For | AAAAAGCAATGGCCGAAATCG | qPCR for *cas9* |
| qcas9 Rev | GGTCAGACGTTGGAATTTCCG | qPCR for *cas9* |
| qrecA For | TGGTGAGATGGGAGCGAGCC | qPCR for *recA* |
| qrecA Rev | TCAGGATTTCCGAACATCACGCC | qPCR for *recA* |
