# Supplementary material for: An Attenuated CRISPR-Cas System in Enterococcus faecalis Permits DNA Acquisition
Source: mBio. 2018 May 1;9(3):e00414-18. doi: 10.1128/mBio.00414-18 (PMC5930301; doi:10.1128/mBio.00414-18)
Supplement: TABLE S3 [file mbo002183850st3.docx]

| **Organism** | | **Strain Name** | | **Description** | **Ref** |
| --- | --- | --- | --- | --- | --- |
| ***E. coli*** | | EC1000 | | *E. coli* cloning host, providing *repA in trans.* F- , *araD139 (ara ABC-leu)7679, galU, galK, lacX74, rspL, thi, repA* of pWV01 in *glgB, km* | (52) |
| ***E. faecalis*** | | V583 | | MDR bloodstream isolate. Van^R^, Gent^R^, Erm^R^ | (6) |
|  | | V649 | | V583 + CRISPR1-*cas9/*tracrRNA in the GISE, a neutral integration site on the *E. faecalis* chromosome | (25) |
|  | | V117 | | V649 with CRISPR1-*cas9* under the control of P_bacA_ | This study |
|  | | CK111SSp (pCF10-101) | | Spontaneous streptomycin-resistant derivative of CK111(pCF10-101) | (25, 36) |
|  | | C173 | | CK111SSp(pCF10-101) with *ermB* disrupting the native *cas9* in the CRISPR1-Cas locus. | (25) |
|  | | V200 | | V117 ΔEF3217 | This study |
|  | | OG117 | | OG1RF with P_bacA_-*cas9*  in the GISE | This study |
|  | | V202 | | V200 “CRISPR edited” with pGR­­-*ermB.* Erm^S^ | This study |
|  | | V204 | | V200 “CRISPR edited” with pGR­­-*ermB.* Erm^S^, Gent^S^ | This study |
|  | | ATCC 29212 | | Used to detect infectious phage particles | (55) |
|  | | OG1RF | | Oral commensal isolate | (7) |
|  | | V649 Δ*vanB* | | V649 with a 100bp deletion in *vanB* | This study |
|  | | V117 Δ*vanB* | | V117 with a 100bp deletion in *vanB* | This study |
|  | | V117 Δ*pstB* | | V117 Δ*pstB* | This study |
|  | | V117 Δ*pstSCAB* | | V117 Δ*pstSCAB* | This study |
|  | | V200 Δ*pstSCAB* | | V200 Δ*pstSCAB* | This study |
|  | | V200 + *tetM* | | V200 with *tetM* inserted between EF1866 and EF1867 | This study |
|  | | OG117 Δ*pstSCAB* | | OG117 Δ*pstSCAB* | This study |
|  | |  | |  |  |
|  | **Plasmid** | | **Description** | | **Ref** |
|  | pCR2-*ermB* | | Targets *ermB* on pTEF1 | | (25) |
|  | pSR-*ermB* | | Intermediate plasmid with the *ermB-*targeting spacer under control of P_bacA_, no first CRISPR repeat | | This study |
|  | pGR-*ermB* | | pSR-*ermB* containing *pheS** | | This study |
|  | pGR-*tetM* | | Isogenic to pGR-*ermB* but with a spacer targeting *tetM* | | This study |
|  | pGR-*vanB* | | Isogenic to pGR-*ermB* but with a spacer targeting *vanB* | | This study |
|  | pGR-*met* | | Isogenic to pGR-*ermB* but with a spacer targeting tRNA-met | | This study |
|  | pGR-*IS1216* | | Isogenic to pGR-*ermB* but with a spacer targeting *IS1216* | | This study |
|  | pGR-*IS256* | | Isogenic to pGR-*ermB* but with a spacer targeting *IS256* | | This study |
|  | pGR-NPV1 | | Isogenic to pGR-*ermB* but with a spacer targeting bacteriophage NPV1 | | This study |
|  | pCE-*vanB* | | CRISPR editing construct to delete 100 bp from *vanB* | | This study |
|  | pCE-*pstB* | | CRISPR editing construct to delete *pstB2* | | This study |
|  | pCE-*pstSCAB* | | CRISPR editing construct to delete *pstS2*, *pstA*, *pstC*, *pstB2*, and *pstB* | | This study |
|  | pCE-3217 | | CRISPR editing construct used to delete EF3217 | | This study |
|  | pCE-tetKI | | CRISPR editing construct used to knock in *tetM* | | This study |
|  | pKH12 | | Conjugative cloning vector | | (25) |
|  | pKHS67 | | pKH12 containing protospacer target for S67 | | (25) |
|  | pKHS5 | | pKH12 containing protospacer target for S5 | | This study |
|  | pG19 | | Allelic-exchange vector to knock in *cas9* | | (24) |
|  | pG19-P_bacA_ | | Allelic-exchange vector to knock in P_bacA_-*cas9* | | This study |
